# Comprehensive evaluation of large language models for pediatric pectus excavatum education

**DOI:** 10.3389/fpubh.2026.1990753

**Published:** 2026-10-30

**Authors:** Hao Shi, Qiang Tang, Jing Xiong, Hou-Chiang Tseng, Tao-Hsing Chang, Xiaolong Chen, Linhao Peng, Haifa Hong

**Affiliations:** 1 Department of General Surgery, Shanghai Children's Hospital, School of Medicine, Shanghai Jiao Tong University, Shanghai, China; 2 Department of General Surgery, Changzhou Children's Hospital of Nantong University, Changzhou, Jiangsu, China; 3 Graduate Institute of Digital Learning and Education, National Taiwan University of Science and Technology, Taipei, Taiwan; 4 Department of Computer Science and Information Engineering, National Kaohsiung University of Science and Technology, Kaohsiung, Taiwan; 5 Department of Cardiothoracic Surgery, Shanghai Children's Hospital, School of Medicine, Shanghai Jiao Tong University, Shanghai, China; 6 School of Artificial Intelligence and Data Science, Shanghai Dianji University, Shanghai, China

**Keywords:** children, large language models, patient education, pectus excavatum, quality, readability

## Abstract

**Background:**

Large language models (LLMs) are increasingly used by patients and caregivers to find health information. However, their performance in providing Simplified Chinese education on pediatric pectus excavatum (PE) remains uncertain. This study comprehensively evaluated the responses generated by five mainstream LLMs.

**Methods:**

Thirty PE-related frequently asked questions (FAQs) spanning five clinical domains were collected and submitted in Simplified Chinese to ChatGPT, Claude, DeepSeek, Qwen, and Kimi. Responses were evaluated by pediatric thoracic surgeons from three institutions for accuracy, completeness, and relevance, using a 6-point Likert scale. Readability was assessed using AlphaReadabilityChinese (ARC) 2.0 and the Chinese Readability Index Explorer (CRIE) 3.0. Understandability, actionability, and overall quality were evaluated with the Chinese Patient Education Materials Assessment Tool for Printable Materials (C-PEMAT-P) and the Global Quality Score (GQS). Friedman tests compared the models, followed by Holm-adjusted Wilcoxon signed-rank tests.

**Results:**

All models achieved mean accuracy, completeness, and relevance scores above 4.9 on the 6-point scale, but the leading model differed by dimension. Claude had the highest mean accuracy (5.39 ± 0.28), completeness (5.22 ± 0.22), and GQS (4.80 ± 0.41), while DeepSeek had the highest relevance (5.42 ± 0.27). Qwen was the least readable: its median CRIE grade was 7.0, compared with 5.5 to 6.0 for the other models. Only 23.3% of its responses were at Grades 1–6, compared with 66.7–86.7% for the others. Overall C-PEMAT-P scores also differed (*p* = 0.003), with Kimi showing the highest numerical mean total (12.17 ± 0.75). Qwen combined the highest actionability (2.57 ± 0.50) with the lowest understandability (9.47 ± 0.57) and GQS (3.10 ± 0.71).

**Conclusion:**

Leading LLMs generally provided accurate, complete, and relevant Simplified Chinese answers to common pediatric PE questions, but no model performed best on every dimensions. LLM outputs should therefore be regarded as a supervised educational aid that require expert verification and adaptation to the health literacy of the intended audience, rather than as substitutes for specialist counseling.

## Introduction

Pectus excavatum (PE) is the most common deformity of the anterior chest wall in children, characterized by posterior displacement of the sternum and adjacent costal cartilages (1). Most affected children have a mild, asymptomatic deformity, but clinical presentation varies. Some are distressed by their appearance, avoid activities that expose the chest, or report reduced exercise tolerance. In severe cases, cardiac compression from sternal depression may reduce cardiopulmonary reserve (2, 3). Caregivers may therefore need information about the natural history of PE, clinical and cardiopulmonary assessment, radiation from imaging, supportive care, vacuum bell therapy, surgery, bar removal, and long-term follow-up. This range of questions makes clear, comprehensible, and clinically bounded education part of pediatric PE care.

PE may progress during pubertal growth, so management may span years. Treatment is individualized because no single approach suits every child. The appropriate strategy depends not only on anatomical severity but also on symptoms, psychosocial effects, evidence of cardiopulmonary compression, chest wall compliance, adherence to conservative therapy, surgical risks, and family preferences (4). As child grow and move through different stages of treatment, families may seek explanations before, during, and after specialist consultations.

Large language models (LLMs) offer families a readily accessible source of health information. LLMs can return conversational explanations rapidly and with little effort, but accessibility does not guarantee clinical adequacy. Reviews of healthcare LLM evaluations identify factual errors, omissions, uneven readability and bias (5). In a pediatric evaluation, more than one-quarter of answers contained expert-identified inaccuracies even though many parents perceived them as clear and trustworthy (6). Thus, an answer can be factually plausible yet incomplete, unsafe, difficult to understand, or inappropriately certain when evidence or patient context is limited.

Two recent studies have begun to examine LLM-generated information specifically for PE. Sirzai et al. compared ChatGPT-4o and Gemini 1.5 using 36 frequently searched questions about PE surgery and reported high expert-rated relevance, accuracy, clarity, and completeness, without significant differences between the models (7). However, they also noted that medical terminology could hinder patient understanding and emphasized the need for specialist verification. Oke et al. subsequently evaluated ChatGPT-4o, Gemini 2.0 Pro, and DeepSeek-V3 across 80 patient-facing questions on chest wall deformities including PE (8). Although most responses were accurate and reproducible, performance differed across models and clinical domains. Treatment and follow-up questions produced the greatest error burden, and hallucinations remained present in all three models. Importantly, both studies were conducted in English, included only two or three LLMs, and did not combine clinical accuracy assessment with formal measures of readability, patient-material comprehensibility, and overall educational quality. Their findings therefore cannot establish how leading international and Chinese LLMs perform when providing pediatric PE education in Simplified Chinese.

At a reported prevalence of 0.5–1%, PE affects a substantial number of children in China’s large pediatric population (9). Chinese families increasingly use online sources, and more recently, to conversational artificial intelligence (AI) to learn about a child’s condition. To our knowledge, no published study has systematically compared several leading LLMs for pediatric PE education in Simplified Chinese. To address this gap, this study aimed to systematically evaluate the performance of five LLMs (ChatGPT, Claude, DeepSeek, Qwen, and Kimi) in answering common questions related to PE. These models were selected because they are among the most widely used AI platforms in China and internationally and are accessible to users with different ages and educational backgrounds. The evaluation covered accuracy, completeness, relevance, readability, comprehensibility, and overall quality.

## Methods

### Study design

This observational, cross-sectional, comparative evaluation study assessed patient-education responses in Simplified Chinese generated by five LLMs regarding pediatric PE. Five LLMs were evaluated: Claude Opus 5 with maximum effort, developed by Anthropic (released on July 24, 2026); ChatGPT 5.6 Sol with Extra-high reasoning effort, developed by OpenAI (released on July 9, 2026); DeepSeek V4pro in max thinking mode, developed by Hangzhou DeepSeek AI Basic Technology Research Co., Ltd. (released on April 24, 2026); Qwen 3.7 with thinking enabled, developed by Alibaba Cloud (released on May 21, 2026) and Kimi K3 with maximum effort, developed by Moonshot AI (released on July 16, 2026). Claude, ChatGPT, Kimi, and Qwen were accessed through their desktop applications, whereas DeepSeek was accessed through its web interface. The reasoning settings reported above represent platform-specific enhanced reasoning options available through the respective user interfaces at the time of evaluation. None of the models was queried through an application programming interface (API) because the study aimed to approximate real-world information-seeking behavior by patients and caregivers rather than to conduct a controlled API-level benchmark of the underlying models.

### Question selection and formulation

We conducted structured question harvesting on 4 August 2026. Frequently asked questions (FAQs) covered basic knowledge, symptoms and diagnosis, observation and conservative treatment, surgical treatment, prognosis and precautions, were compiled from three sources:

1. Published international and domestic guidelines and consensus statements (4, 10, 11);
2. Patient-education pages from children’s hospitals and academic medical centers;
3. Patient and caregiver communities; and official Chinese societies and authoritative pediatric hospitals.

One senior attending physician reviewed these materials based on high search frequency, high views or clicks counts, pinned or recommended status, and repeated across sources. To be eligible, questions had to explicitly concern PE in children or adolescents and address one of five predefined domains. Each question also had to be suitable for formulation as a standalone health-related question relevant to caregivers. Ten questions were initially selected from each domain, yielding a candidate pool of 50 questions. Three researchers then reviewed the 50 candidate questions and reached consensus through discussion. They excluded questions that were duplicates, overlapped substantially in meaning, or were too specialized or medically detailed for general patient education. They also excluded questions that required individualized examinations or other patient-specific information for an appropriate answer. After screening, 30 FAQs were retained for subsequent evaluation of LLMs (Table 1).

**Table 1:** FAQs related to pediatric PE.

FAQs related to pediatric PE
A. Basic knowledge
What is pediatric pectus excavatum?
When does pectus excavatum usually become apparent?
Who is more likely to develop pectus excavatum?
Is pectus excavatum hereditary?
What causes pectus excavatum?
Can pectus excavatum resolve on its own?
B. Symptoms and diagnosis
What types of pectus excavatum are there?
Besides a sunken chest, what other symptoms can pectus excavatum cause?
Can pectus excavatum affect the heart and lungs?
What examinations are usually needed to evaluate pectus excavatum?
What are the Haller index and correction index?
What is cardiopulmonary exercise testing?
C. Observation and conservative treatment
What treatments are available for pectus excavatum?
Can posture training and breathing exercises help children with pectus excavatum?
Which children are suitable candidates for vacuum bell therapy?
How should a vacuum bell be used?
How effective is vacuum bell therapy for pectus excavatum?
What adverse effects can occur with vacuum bell therapy?
D. Surgical treatment
What are the indications of surgery for children with pectus excavatum?
At what age is surgery for pediatric pectus excavatum usually performed?
What surgical procedures are available for pectus excavatum?
What is the Nuss procedure?
Is the Nuss procedure minimally invasive?
What complications can occur after pectus excavatum surgery?
E. Prognosis and precautions
Does the bar placed during the Nuss procedure need to be removed, and when is it usually removed?
Can a child with a pectus bar undergo X-rays, CT scans, and MRI scans?
When can a child resume daily activities and exercise after pectus excavatum surgery?
How should a child be followed up after pectus excavatum surgery?
How effective is pectus excavatum surgery?
Can pectus excavatum recur after surgery?

All questions were submitted in Simplified Chinese using an identical standardized prompt. Each question was entered into a separate, newly initiated, context-free conversation to prevent information carryover between questions. To preserve a natural conversational question-and-answer format, the models were instructed not to provide in-text citations, reference lists, source names, or hyperlinks. Web-browsing, internet-search, retrieval-augmented generation, deep-research were disabled throughout the evaluation. The models were instructed to respond directly to pediatric patients with PE and their caregivers using clear, accessible, accurate, and objective language and to limit each response to no more than 300 Chinese characters. The character count included Chinese characters, numbers, English letters, and punctuation marks. Full prompt text provided in Supplementary material. All queries and response collection were completed on 6 August 2026.

### Ethical considerations

All data analyzed in this study were generated by LLMs. The study involved neither human participants nor animals and did not use any personally identifiable information or human-derived biological specimens. Accordingly, under applicable international and Chinese research ethics guidelines, institutional ethics committee review and approval were not required.

### Expert evaluation

Nine pediatric thoracic surgeons from three institutions formed the evaluation panel: six from Shanghai Children’s Hospital, two from Changzhou Children’s Hospital, and one from Shanghai Sixth People’s Hospital. Each had 5–10 years of clinical experience in pediatric PE. Reviewers were blinded to model identity, and the responses were anonymized and presented in random order to reduce potential model-identification and ordering effects. Before the formal evaluation, we established key points for each question based on relevant clinical guidelines and consensus recommendations for pediatric PE. These key points served as a standardized reference for evaluating the responses.

#### Accuracy, completeness, and relevance analysis

Six experts (three from Shanghai Children’s Hospital, one from Shanghai Sixth People’s Hospital and two from Changzhou Children’s Hospital) independently rated every response for accuracy, completeness, and relevance.

Accuracy was defined as the extent to which the LLM-generated response was factually correct, precise, internally consistent, and free from substantive errors (12). Completeness was defined as the extent to which the response addressed all essential aspects of the user’s query and provided an appropriately comprehensive overview or sufficient detail (12). Relevance was defined as the extent to which the response directly addressed the user’s query while avoiding unnecessary, redundant, or unrelated information (12).

Each dimension was rated independently using a 6-point Likert scale ranging from 1 to 6, with higher scores indicating better performance (Table 2).

**Table 2:** Likert score scale of accuracy, completeness, and relevance.

Items	Points	Definition
Accuracy	1	Completely inaccurate, with serious errors
	2	Mostly inaccurate, with several obvious errors
	3	Partially accurate, but contains important errors
	4	Generally accurate, with only a few errors
	5	Highly accurate, with only minor flaws
	6	Completely accurate, with no evident errors
Completeness	1	Completely incomplete and barely addresses the question
	2	Mostly incomplete, with most key information missing
	3	Partially complete, with several important points missing
	4	Generally complete, but some important information is missing
	5	Highly complete, with only minor information omitted
	6	Fully complete and covers all key information
Relevance	1	Completely irrelevant and does not address the question
	2	Mostly irrelevant, with most content unrelated to the question
	3	Partially relevant, but contains substantial off-topic content
	4	Generally relevant, with some unnecessary information
	5	Highly relevant, with almost no irrelevant information
	6	Completely relevant, with all content directly addressing the question

#### Readability analysis

Each of the responses was saved as a separate UTF-8 plain-text file. Model identifiers and platform metadata were excluded, whereas the substantive wording, punctuation, paragraph breaks, and model-generated list structure were retained.

Readability was assessed using AlphaReadabilityChinese (ARC) 2.0,^1^ which generated ten published lexical, syntactic, semantic and coherence indices (13). Higher lexical richness, syntactic richness, noun semantic richness, and noun semantic noise are associated with greater reading difficulty, whereas higher values for the four semantic precision indices, noun semantic clarity, and coherence generally reflect greater semantic clarity and textual comprehensibility.

Responses were also analyzed using the native-Chinese-reader module of the Chinese Readability Index Explorer (CRIE) 3.0^2^ (14). CRIE estimates a grade level from 1 to 12, with higher grades indicating more difficult text. Grades 1–6 represent elementary school, grades 7–9 middle school, and grades 10–12 high school.

#### Understandability, actionability, and overall quality analysis

Two experts independently assessed all responses using the Chinese Patient Education Materials Assessment Tool for Printable Materials (C-PEMAT-P) and the Global Quality Score (GQS). For disagreements in C-PEMAT-P item ratings or GQS scores, the third expert reviewed the corresponding response and adjudicated the final rating after discussion.

The original C-PEMAT-P consists of 24 items across two domains: understandability (17 items assessing logical organization and the accessibility of language and terminology) and actionability (7 items assessing the clarity of recommended actions and their suitability for the intended audience) (15). The original C-PEMAT-P was developed for conventional patient education materials. For short conversational LLM responses, we used a prespecified, study-specific 14-item adaptation to assess understandability and actionability on an exploratory basis. Each response was rated as a stand-alone text intended for patients. Before rating began, we excluded 10 items that required formats or functions absent from all responses by design, including images, tables, calculation examples, and tools for planning or recording. The adapted form contained 11 understandability items and 3 actionability items, with a maximum score of 14. Reviewers used the same fixed form for every response and did not assess item applicability individually. We removed the N/A option to keep the item set and scoring denominator the same across all models. This adaptation has not been independently validated and should not be considered equivalent to the original 24-item C-PEMAT-P.

The GQS was used to assess the overall quality of the LLM-generated responses (16). It uses a 5-point scale: 1 indicates poor quality, with disorganized content, missing key information, and no practical value; 2 indicates generally poor quality, with weak logical structure, incomplete core information, and limited practical value; 3 indicates moderate quality, with some important information adequately addressed and basic practical value; 4 indicates good quality, with clear logical structure, coverage of most relevant information, and relatively high practical value; and 5 indicates excellent quality, with rigorous logic, coherent presentation, and high practical value for users.

#### Statistical analysis

Statistical analyses and visualizations were performed using Python 3.12. For each question–model pair, the ratings were averaged separately before statistical testing. Questions served as matched blocks across the five models, yielding 30 paired observations per model for overall analyses and six paired observations per model for domain-specific analyses. Continuous variables are reported as mean ± SD, while ordinal or non-normally distributed variables are reported as median (IQR). Tie-corrected Friedman tests assessed overall and domain-specific differences among models. Inter-rater agreement was assessed using a two-way random-effects, absolute-agreement intraclass correlation coefficient (ICC) for accuracy, completeness, and relevance and Cohen’s kappa for C-PEMAT-P and GQS score, with κ values > 0.75 considered indicative of excellent agreement, values between 0.40 and 0.75 indicating acceptable agreement, and values < 0.40 indicating poor agreement (17, 18).

For overall outcomes, Holm adjustment was applied separately across accuracy, completeness, and relevance, the 10 ARC metrics, C-PEMAT-P tests comprising total score, understandability, and actionability. The omnibus *p* values for overall CRIE grade and GQS were reported without cross-outcome adjustment. Pairwise model comparisons used two-sided exact Wilcoxon signed-rank tests, with zero differences excluded from the signed-rank calculations. Holm adjustment was applied across the 10 model-pair comparisons separately within each outcome. For domain-specific analyses, omnibus *p* values were Holm-adjusted across the five clinical domains separately for each outcome. *p* < 0.05 indicated statistical significance.

## Results

### Accuracy, completeness, and relevance analysis

All five LLMs received generally high expert ratings, with mean scores above 4.9 on the 6-point scale. The models nevertheless differed in accuracy (χ^2^ = 14.14, *p* = 0.010), completeness (χ^2^ = 14.75, *p* = 0.010), and relevance (χ^2^ = 40.02, *p* < 0.001) (Supplementary Table S1). Claude had the highest mean accuracy (5.39 ± 0.28) and completeness (5.22 ± 0.22), while DeepSeek had the highest mean relevance (5.42 ± 0.27). Pairwise comparisons showed higher accuracy for Claude than for ChatGPT (*p* = 0.019), DeepSeek (*p* = 0.019), Kimi (*p* < 0.001), and Qwen (*p* = 0.038) (Supplementary Table S2; Figure 1). Claude also had higher completeness than ChatGPT (*p* = 0.002), with no other pairwise difference in completeness was significant. Qwen had the lowest relevance (4.94 ± 0.37), scoring 0.33–0.48 points below each of the other four models (*p* ≤ 0.001). No other pairwise difference in relevance was significant.

**Figure 1:**
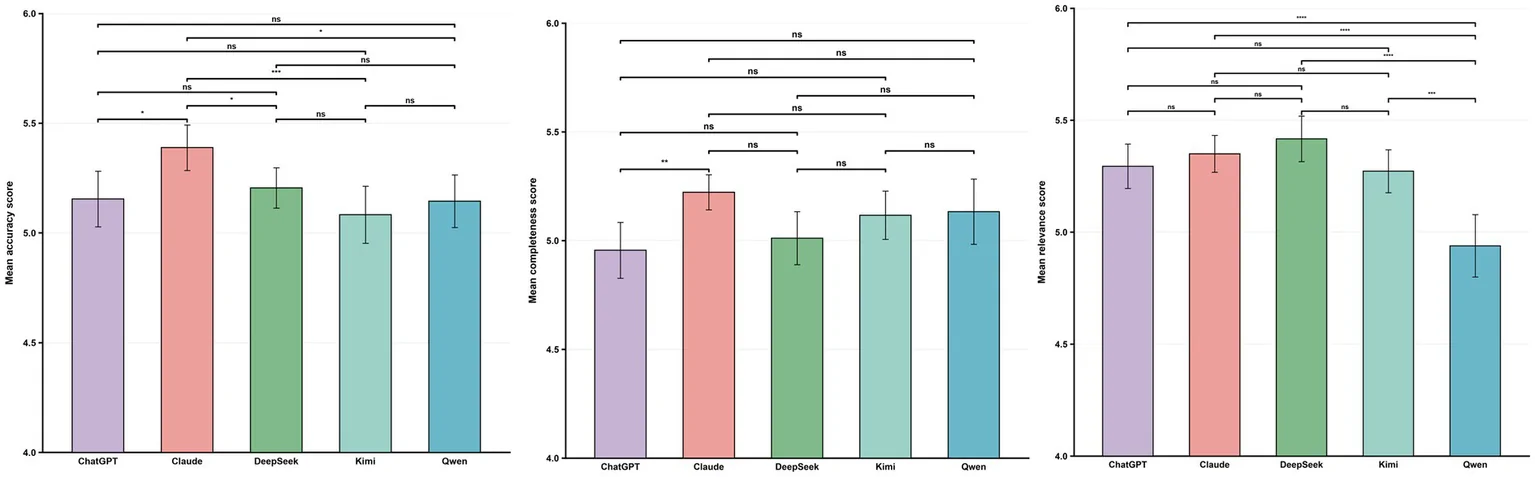
Overall evaluation of accuracy, completeness and relevance in five LLMs.

Figure 2 presents domain-level results for basic knowledge, symptoms and diagnosis, observation and conservative treatment, surgical treatment, and prognosis and precautions. Supplementary Table S3 provides the numerical values. Accuracy and completeness did not differ significantly among models in any domain. Claude had the highest mean accuracy in four of five domains, while completeness scores largely overlapped. Relevance differed only for basic knowledge (χ^2^ = 13.47, *p* = 0.037) and observation and conservative treatment (χ^2^ = 14.41, *p* = 0.030). DeepSeek had the highest mean relevance for basic knowledge, observation and conservative treatment, and prognosis and precautions. Qwen had the lowest mean relevance in all five domains. Supplementary Figure S1 presents question-level results. The six surgeons demonstrated moderate overall inter-rater reliability for the clinical content assessments. The average-measure two-way random ICCs (2, 6) were 0.55 (95% CI, 0.24–0.76) for accuracy, 0.52 (95% CI, 0.19–0.74) for completeness, and 0.69 (95% CI, 0.49–0.83) for relevance.

**Figure 2:**
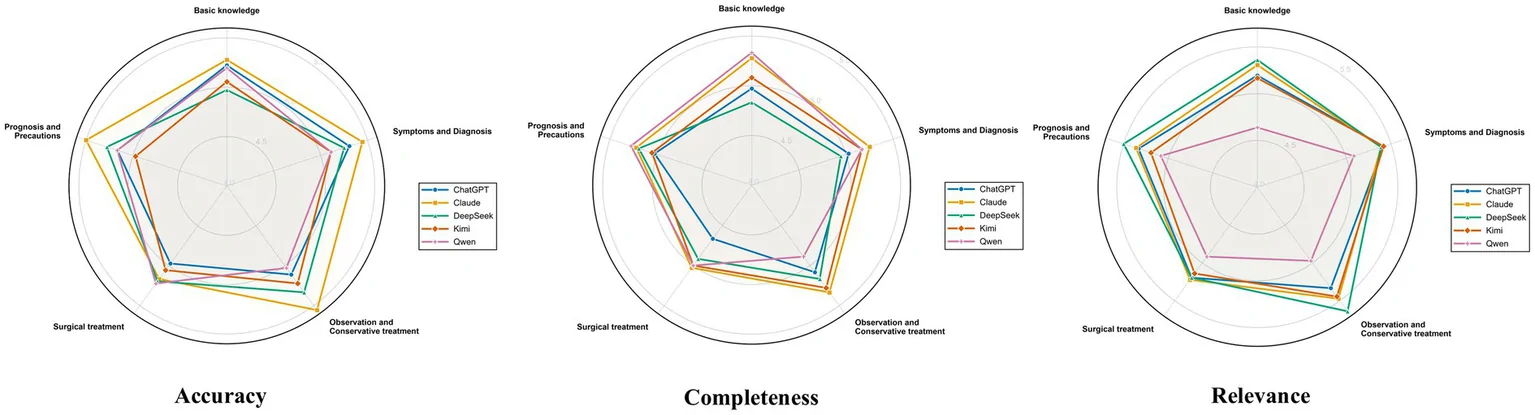
Accuracy, completeness and relevance across domains in five LLMs.

### Readability analysis

#### CRIE

CRIE grade levels differed among the five LLMs (χ^2^ = 43.14, *p* < 0.001; Supplementary Table S4). Median grades were 5.5 (IQR, 3.0–6.0) for ChatGPT, 6.0 (4.0–6.0) for Claude, 6.0 (4.0–7.0) for DeepSeek, 6.0 (5.0–6.0) for Kimi, and 7.0 (7.0–8.0) for Qwen. Because higher grades indicate more difficult text, Qwen produced the least readable responses. Its grades were significantly higher than those of every other model (*p* < 0.001) (Figure 3), with no significant differences among ChatGPT, Claude, DeepSeek, and Kimi (Supplementary Table S5). Grade distributions showed the same pattern. Most responses from Kimi (86.7%), ChatGPT (80.0%), Claude (76.7%), and DeepSeek (66.7%) were at Grades 1–6. Only 23.3% of Qwen responses fell within this range, while 76.7% were at Grades 7–9. Grades 10–12 occurred in only one response each from ChatGPT and DeepSeek (Figure 3).

**Figure 3:**
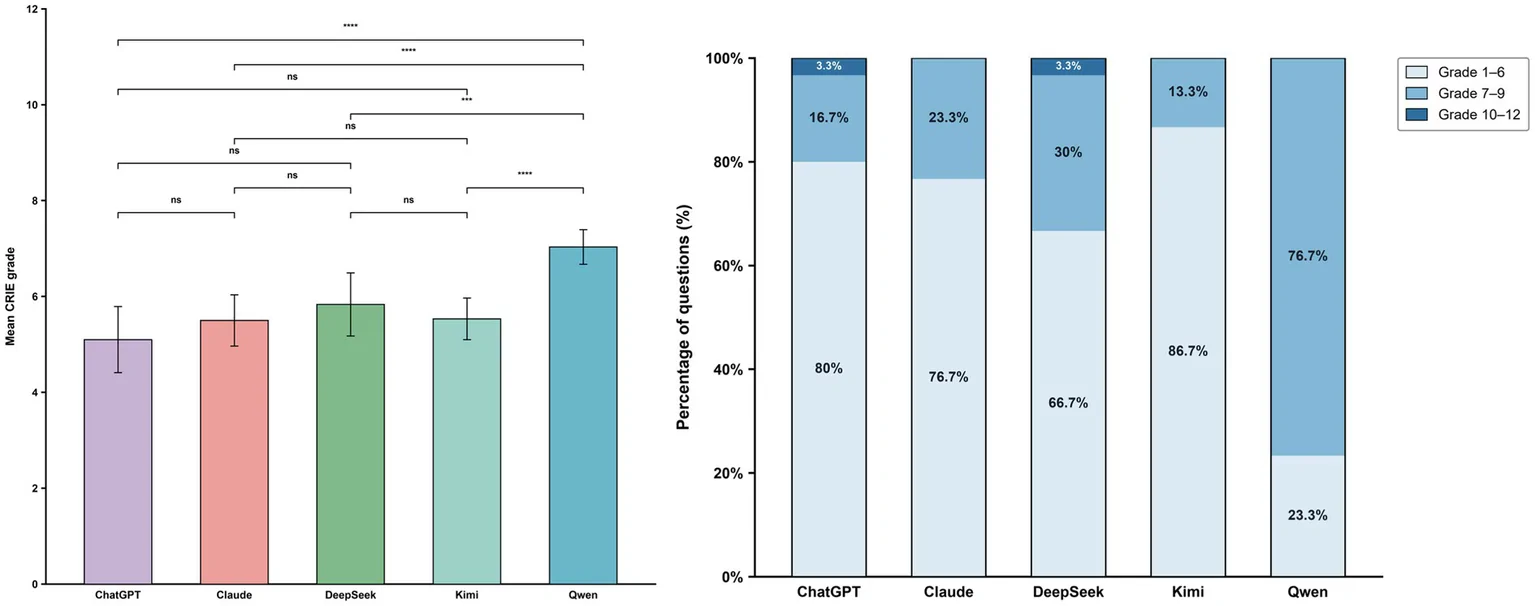
Overall evaluation of CRIE grade and their distribution in five LLMs.

Grade levels differed by model for basic knowledge (χ^2^ = 15.50, *p* = 0.019) and prognosis and precautions (χ^2^ = 14.46, *p* = 0.024), but not for symptoms and diagnosis, observation and conservative treatment and surgical treatment (Supplementary Table S6). All ChatGPT and Claude responses in basic knowledge were at Grades 1–6, compared with 50.0% of Qwen responses (Figure 4). For observation and conservative treatment, all ChatGPT and Kimi responses were at Grades 1–6, while 66.7% of Qwen responses were at Grades 7–9. The largest contrast occurred in prognosis and precautions: every Qwen response was at Grades 7–9, compared with 83.3–100% of responses from the other models at Grades 1–6. Qwen’s greater reading difficulty therefore appeared in several clinical domains.

**Figure 4:**
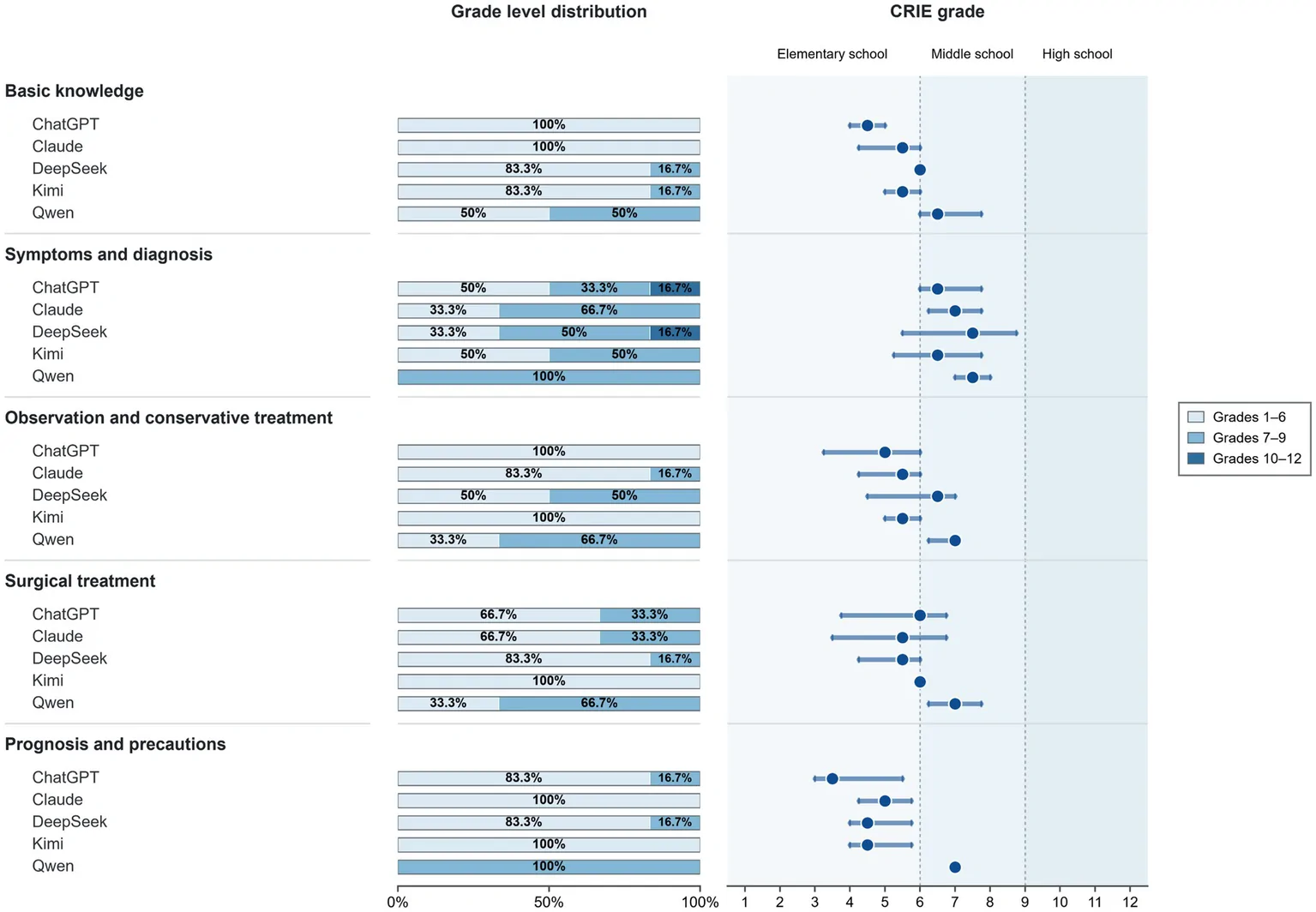
CRIE grade levels across clinical domains in five LLMs.

#### ARC

Eight of ten ARC metrics differed among the five LLMs, including lexical richness, syntactic richness, noun semantic accuracy, noun–verb semantic accuracy, content-word semantic accuracy, noun semantic richness, noun semantic noise, and coherence (Table 3). Verb semantic accuracy (χ^2^ = 9.60, *p* = 0.095) and noun semantic clarity (χ^2^ = 3.33, *p* = 0.504) did not differ significantly.

**Table 3:** Overall comparison of ARC readability metrics among the five LLMs.

Metrics, M(Q1, Q3)	ChatGPT	Claude	DeepSeek	Kimi	Qwen	Kendall’s W (95% CI)	χ^2^	Adjusted *p*
Lexical richness	3.99 (3.91, 4.06)	4.54 (4.48, 4.57)	4.32 (4.22, 4.46)	4.56 (4.44, 4.66)	4.97 (4.79, 5.11)	0.84 (0.78–0.91)	101.01	**<0.001** ^*^
Syntactic richness	1.96 (1.90, 2.01)	2.02 (1.98, 2.06)	2.00 (1.96, 2.06)	2.06 (2.02, 2.10)	2.04 (2.02, 2.09)	0.31 (0.19–0.48)	36.72	**<0.001** ^*^
Semantic accuracy-N	2.92 (2.47, 3.36)	3.66 (3.29, 4.08)	3.11 (2.61, 3.65)	3.93 (3.62, 4.17)	3.52 (3.37, 3.83)	0.34 (0.18–0.57)	40.90	**<0.001** ^*^
Semantic accuracy-V	6.26 (5.78, 6.63)	6.29 (5.99, 6.99)	6.04 (5.72, 6.57)	6.32 (5.91, 7.07)	6.46 (6.31, 6.84)	0.08 (0.04–0.21)	9.60	0.095
Semantic accuracy-N-V	4.87 (4.12, 5.20)	5.22 (4.82, 5.51)	4.70 (4.44, 5.01)	5.33 (5.12, 5.59)	5.23 (5.05, 5.61)	0.27 (0.15–0.46)	31.87	**<0.001** ^*^
Semantic accuracy-content words	4.92 (4.38, 5.32)	5.26 (4.94, 5.59)	4.88 (4.47, 5.20)	5.26 (5.04, 5.53)	5.20 (5.04, 5.52)	0.20 (0.09–0.39)	24.03	**<0.001** ^*^
Semantic richness-N	0.26 (0.23, 0.31)	0.25 (0.21, 0.27)	0.26 (0.22, 0.30)	0.24 (0.21, 0.26)	0.26 (0.23, 0.28)	0.17 (0.08–0.33)	20.03	**0.002** ^*^
Semantic clarity-N	0.004 (0.003, 0.006)	0.004 (0.003, 0.005)	0.003 (0.002, 0.006)	0.004 (0.003, 0.005)	0.003 (0.003, 0.005)	0.03 (0.01–0.16)	3.33	0.504
Semantic noise-N	5.41 (3.25, 8.52)	9.84 (5.58, 10.82)	7.96 (4.02, 12.32)	7.55 (5.49, 10.41)	10.14 (8.43, 12.14)	0.15 (0.08–0.31)	18.24	**0.003** ^*^
Coherence	0.79 (0.77, 0.82)	0.77 (0.76, 0.78)	0.78 (0.75, 0.82)	0.78 (0.76, 0.81)	0.75 (0.73, 0.77)	0.25 (0.13–0.45)	30.11	**<0.001** ^*^

Bold and * indicate statistically significant differences (*p* < 0.05).

*Post hoc* comparisons showed that Qwen had the highest lexical richness and the lowest coherence, with both measures differing from every other model, whereas ChatGPT had significantly lower syntactic richness than the other four models (Supplementary Table S7). Differences in semantic-accuracy metrics occurred mainly between ChatGPT or DeepSeek and the other models (Figure 5).

**Figure 5:**
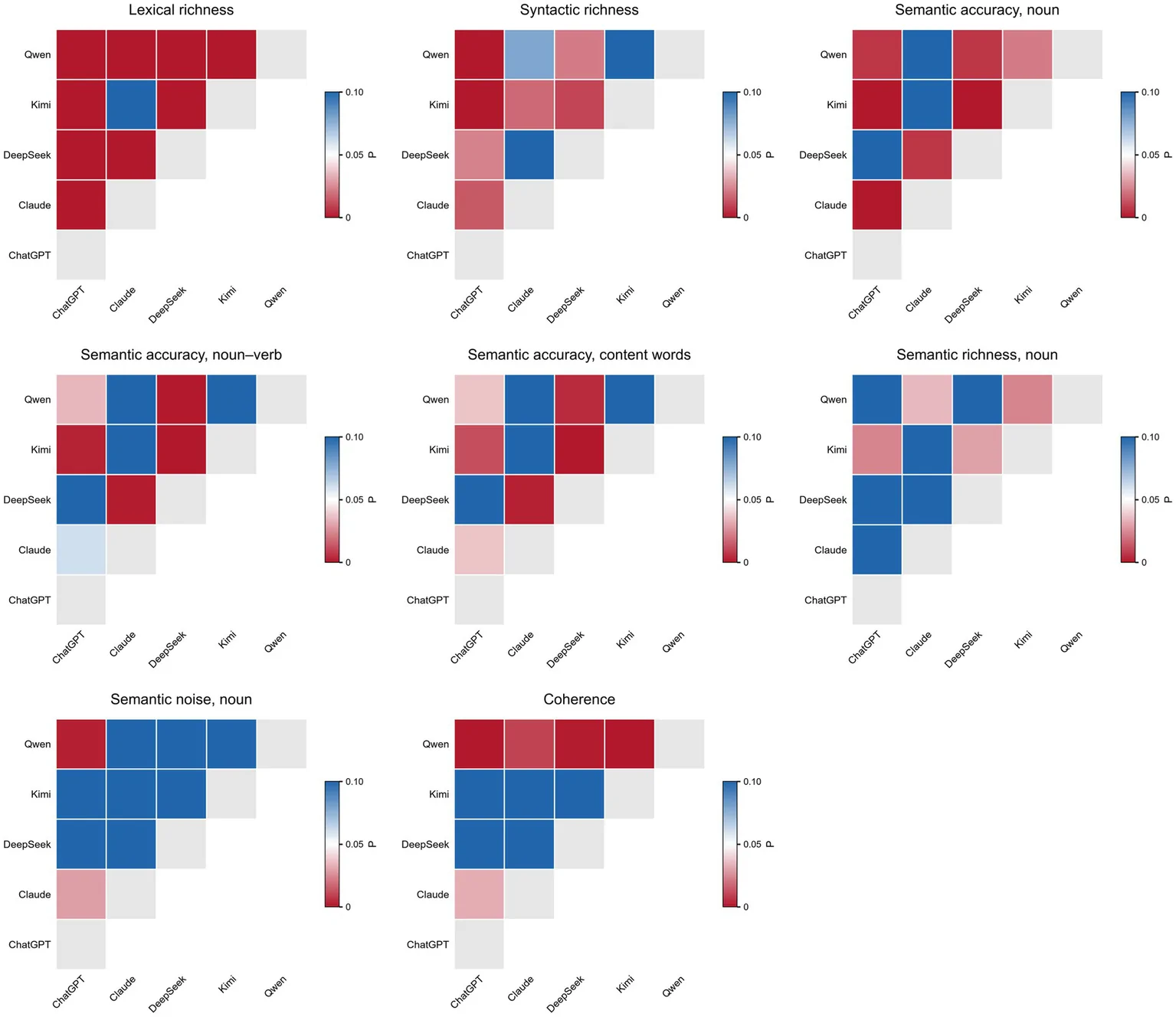
Heatmap of pairwise comparisons in ARC readability metrics across five LLMs.

### C-PEMAT-P and overall quality analysis

GQS scores differed among models (χ^2^ = 78.74, *p* < 0.001; Table 4). Claude scored highest (4.80 ± 0.41), followed by ChatGPT (4.23 ± 0.43), Kimi (4.00 ± 0.69), DeepSeek (3.80 ± 0.55), and Qwen (3.10 ± 0.71). Claude scored higher than every other LLM, while Qwen scored lower than every other LLM (Supplementary Table S8). ChatGPT also scored higher than DeepSeek. Differences between ChatGPT and Kimi and between DeepSeek and Kimi were not significant (Figure 6).

**Table 4:** C-PEMAT-P and GQS scores across the five LLMs.

Metrics	ChatGPT	Claude	DeepSeek	Kimi	Qwen	Kendall’s W (95% CI)	χ^2^	Adjusted *p*
C-PEMAT-P	11.37 ± 1.22	11.87 ± 0.94	11.83 ± 0.95	12.17 ± 0.75	12.03 ± 0.67	0.13 (0.07–0.28)	15.85	0.003*
C-PEMAT-P understandability	9.90 ± 0.31	10.00 ± 0.00	10.07 ± 0.25	10.10 ± 0.31	9.47 ± 0.57	0.39 (0.19–0.60)	46.26	<0.001*
C-PEMAT-P actionability	1.47 ± 1.11	1.87 ± 0.94	1.77 ± 0.97	2.07 ± 0.69	2.57 ± 0.50	0.34 (0.20–0.51)	40.50	<0.001*
GQS	4.23 ± 0.43	4.80 ± 0.41	3.80 ± 0.55	4.00 ± 0.69	3.10 ± 0.71	0.66 (0.55–0.76)	78.74	<0.001* ^**,†**^

^†^The GQS *p* value is unadjusted. Bold and * indicate statistically significant differences (*p* < 0.05).

**Figure 6:**
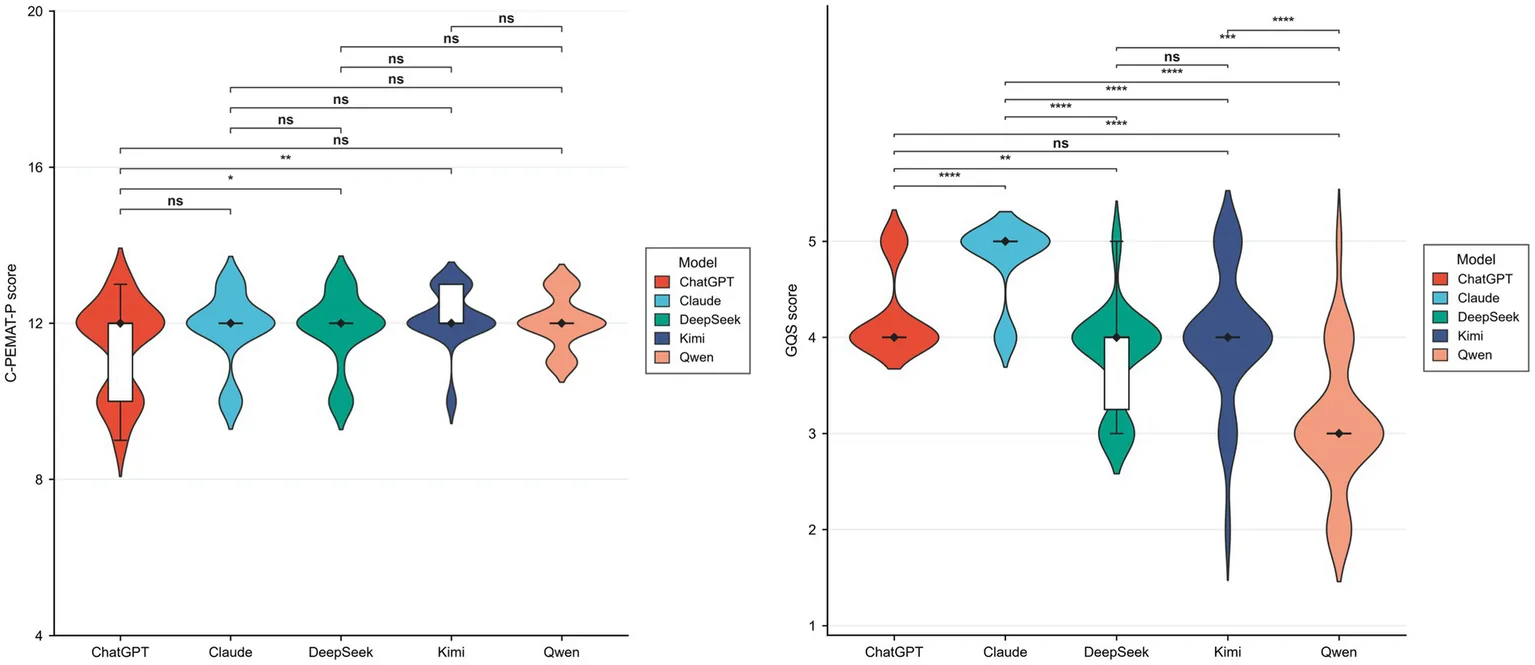
C-PEMAT-P and GQS scores of five LLMs.

Total C-PEMAT-P scores also differed among the five LLMs (χ^2^ = 15.85, *p* = 0.003; Table 4). Mean scores were 12.17 ± 0.75 for Kimi, 12.03 ± 0.67 for Qwen, 11.87 ± 0.94 for Claude, 11.83 ± 0.95 for DeepSeek, and 11.37 ± 1.22 for ChatGPT. ChatGPT scored lower than DeepSeek and Kimi in *post hoc* comparisons; the remaining pairwise differences were not significant (Figure 6).

Both C-PEMAT-P subscales differed among models (understandability: χ^2^ = 46.26; actionability: χ^2^ = 40.50; both *p* < 0.001). Kimi had the highest understandability score (10.10 ± 0.31), and Qwen had the lowest (9.47 ± 0.57) (Table 4). Qwen scored lower on understandability than every other model; no other pairwise difference was significant (Supplementary Table S9). Qwen had the highest actionability score (2.57 ± 0.50), and ChatGPT had the lowest (1.47 ± 1.11) (Table 4). Qwen scored higher on actionability than every other model, and Kimi scored higher than ChatGPT. No other pairwise difference was significant (Supplementary Table S9).

At the domain level, the between-model comparison of total C-PEMAT-P scores for basic knowledge yielded χ^2^ = 13.29, Kendall’s W = 0.55 (95% CI, 0.31–0.89), and a Holm-adjusted *p* value of 0.0498 (Table 5). Although this result met the specified the threshold, the six-question domain sample and the absence of study-wide multiplicity control warrant caution. Kimi and Qwen shared the highest mean in this domain (12.33 ± 0.52). The comparisons did not reach the threshold for symptoms and diagnosis (χ^2^ = 6.35, *p* = 0.349), observation and conservative treatment (χ^2^ = 8.64, *p* = 0.212), surgical treatment (χ^2^ = 3.02, *p* = 0.554), or prognosis and precautions (χ^2^ = 10.00, *p* = 0.162). Qwen had the highest numerical mean score for symptoms and diagnosis, while Claude and DeepSeek had the highest numerical means for observation and conservative treatment.

**Table 5:** C-PEMAT-P and GQS scores across domains in five LLMs.

Clinical domain	ChatGPT	Claude	DeepSeek	Kimi	Qwen	Kendall’s W (95% CI)	χ^2^	Adjusted P
C-PEMAT-P								
Basic Knowledge	10.50 ± 1.38	11.33 ± 1.03	11.17 ± 0.98	12.33 ± 0.52	12.33 ± 0.52	0.55 (0.31–0.89)	13.29	0.0498*
Symptoms and Diagnosis	10.33 ± 0.82	11.00 ± 1.10	11.33 ± 1.03	11.33 ± 1.03	11.83 ± 0.75	0.26 (0.07–0.77)	6.35	0.349
Observation and Conservative Treatment	12.17 ± 0.41	12.50 ± 0.55	12.50 ± 0.55	12.33 ± 0.52	11.83 ± 0.75	0.36 (0.17–0.73)	8.64	0.212
Surgical Treatment	11.33 ± 1.03	12.00 ± 0.00	11.67 ± 0.82	12.00 ± 0.00	12.00 ± 0.63	0.13 (0.04–0.58)	3.02	0.554
Prognosis and Precautions	12.50 ± 0.55	12.50 ± 0.55	12.50 ± 0.55	12.83 ± 0.41	12.17 ± 0.75	0.42 (0.21–0.79)	10	0.162
GQS								
Basic Knowledge	4.00 ± 0.00	4.50 ± 0.55	3.50 ± 0.55	3.83 ± 0.41	2.50 ± 0.55	0.81 (0.78–0.92)	19.40	**0.003** ^*^
Symptoms and Diagnosis	4.33 ± 0.52	5.00 ± 0.00	3.83 ± 0.41	4.00 ± 1.10	3.50 ± 1.05	0.50 (0.37–0.84)	12.09	**0.017** ^*^
Observation and Conservative Treatment	4.00 ± 0.00	4.67 ± 0.52	3.67 ± 0.52	4.00 ± 0.63	3.17 ± 0.41	0.66 (0.41–0.91)	15.95	**0.006** ^*^
Surgical Treatment	4.33 ± 0.52	5.00 ± 0.00	3.83 ± 0.41	3.83 ± 0.75	3.33 ± 0.52	0.75 (0.68–0.90)	18.06	**0.005** ^*^
Prognosis and Precautions	4.50 ± 0.55	4.83 ± 0.41	4.17 ± 0.75	4.33 ± 0.52	3.00 ± 0.63	0.73 (0.69–0.89)	17.46	**0.005** ^*^

Bold and * indicate statistically significant differences (*p* < 0.05).

GQS scores differed among models in every domain: basic knowledge (χ^2^ = 19.40, *p* = 0.003), symptoms and diagnosis (χ^2^ = 12.09, *p* = 0.017), observation and conservative treatment (χ^2^ = 15.95, *p* = 0.006), surgical treatment (χ^2^ = 18.06, *p* = 0.005), and prognosis and precautions (χ^2^ = 17.46, *p* = 0.005) (Table 5). Claude had the highest mean GQS score in every domain, and Qwen had the lowest. The overall inter-rater agreement was acceptable in C-PEMAT-P and excellent in GQS score. Mean pairwise unweighted Cohen’s κ was 0.61 for C-PEMAT-P (95% CI, 0.40–0.81) and 0.82 for GQS score (95% CI, 0.65–0.98).

## Discussion

We evaluated five LLMs as sources of Simplified Chinese patient education about pediatric PE. Blinded experts from several centers assessed clinical content, while Chinese-language instruments measured readability and the suitability of patient-education material. The five models generally produced medically sound answers, but they differed in accuracy, completeness, relevance, readability, understandability, actionability, and overall quality. No model led on every measure, and strength on one measure did not reliably predict strength on another. Accurate information could still be difficult for patients and caregivers to understand or use. To our knowledge, this is the first comparison of leading international and Chinese LLMs for pediatric PE education in Simplified Chinese and the first to use readability tools developed and validated specifically for Chinese text rather than formulas derived from English.

All five LLMs scored highly for accuracy, completeness, and relevance, in line with earlier studies of patient-facing PE responses (7, 8). These measures did not identify one leading model. Claude had the highest mean accuracy and completeness, although few pairwise differences in completeness reached significance, and DeepSeek ranked highest relevance. Qwen had relatively high completeness scores for basic knowledge and for prognosis and precautions, but it had the lowest mean relevance in every domain and scored below the other four models overall. Greater coverage did not always produce a more focused answer. Patient-facing FAQs need to address the immediate question while retaining essential qualifications, not reproduce the breadth of a textbook summary. The character limit may have intensified the trade-off between completeness and focus: omitting key points reduces clinical adequacy, while excessive detail can hide the main message. Accuracy is necessary, but completeness and relevance determine whether correct information is sufficiently informative and focused. High average scores also do not rule out individual errors or omissions that matter clinically.

Similar trade-offs have been reported in other researches. Keçeci et al. compared ChatGPT-4o and DeepSeek using questions derived from the AAOS clinical practice guideline for clavicle fracture management (19). Both models achieved generally high accuracy, but occasional inaccuracies and hallucinations remained. Neither produced meaningful PEMAT actionability scores, and DeepSeek’s longer responses did not result in better overall quality. In a study more directly relevant to patient education, Karagoz et al. compared ChatGPT-5 and DeepSeek V3 across 35 foot and ankle disorders using DISCERN, PEMAT-P, GQS, CLEAR, and readability measures (20). ChatGPT-5 performed better in reliability, patient-education quality, and overall content quality, whereas DeepSeek V3 produced more readable text. These findings are consistent with the variation we observed within individual models across clinical accuracy, readability, understandability, actionability, and overall quality. They suggest that factual or guideline-level accuracy alone is insufficient to establish whether LLM-generated information is suitable for patient education. Readability, understandability, and actionability each require separate evaluation.

CRIE and ARC described different aspects of the same readability pattern. Qwen had the highest estimated CRIE grade, and 76.7% of its responses were at Grades 7–9. Most responses from the other models remained at Grades 1–6, making Qwen’s text the most difficult. ARC likewise found the highest lexical richness, lowest coherence, and relatively high noun semantic noise for Qwen. Dense vocabulary and weaker continuity may partly explain its higher grade level. Yet Qwen also performed well on several semantic-accuracy indices, so semantic precision did not necessarily make the response easier to read. ChatGPT showed the reverse pattern: lower lexical and syntactic richness and higher coherence accompanied a lower CRIE grade. Used together, CRIE and ARC show why patient-facing responses need familiar vocabulary, coherent organization, and concise explanations rather than technical density alone.

Total C-PEMAT-P scores covered a narrow range, from 11.37 ± 1.22 for ChatGPT to 12.17 ± 0.75 for Kimi. These values should be interpreted against the adjusted maximum of 14, not the nominal 24, because items requiring images, tables, or planning tools did not apply to text-only responses. All models reached 81–87% of the applicable maximum. Most variation occurred in actionability. Understandability scores were near the ceiling, with only Qwen differing significantly from the other models, while actionability ranged from 1.47 ± 1.11 for ChatGPT to 2.57 ± 0.50 for Qwen. Actionability findings should be interpreted cautiously. The adapted form contained only three actionability items, and many FAQs sought information without necessarily calling for action. A question about the Haller index, for example, does not inherently require a recommended action. All models were assessed using the same questions and scoring framework. However, differences in actionability may partly reflect question type and should not be interpreted as differences in intrinsic model capability. GQS separated the models more clearly and produced a different ranking. Claude ranked highest and Qwen lowest across every domain, with each differing from all other models. ChatGPT produced the simplest and most cohesive text but had the lowest total C-PEMAT-P and actionability, whereas Qwen combined the highest actionability with the most difficult text and the lowest understandability and GQS. Overall quality, understandability, and actionability measure different properties, relying on one instrument may misclassify a model’s suitability for patient education.

The study directly compared LLMs developed internationally and in China. Claude and ChatGPT ranked first and second on GQS even though every question and responses was in Simplified Chinese, so models developed in China showed no consistent advantage in this setting. Chinese-language benchmarks in tuberculosis, pertussis, and skin cancer have likewise reported performance that depends on the model and metric rather than a fixed hierarchy. ChatGPT perform well on selected measures of educational suitability, overall quality, or factual accuracy (17, 18, 21). Results also vary among PE studies. Oke et al. found that DeepSeek-V3 had the highest hallucination rate among the models they tested (8). Models developed in China were not uniformly poorer in our data. DeepSeek had the highest relevance, and Qwen had the highest actionability. Kimi and Qwen have not previously been evaluated for PE education in any language. Selecting a model for Chinese pediatric patient education should therefore depend on the intended audience and the dimensions that matter for the task, not on where the model was developed.

Patients and caregivers increasingly use accessible AI to obtain health information, often before a hospital visit. This pattern may be particularly relevant in China’s high-volume healthcare system, where large patient numbers and busy outpatient services can limit time for detailed, individualized explanations. During a clinical encounter, LLMs may supplement education about the Haller index, treatment options, timing of intervention, and perioperative issues, including the Nuss procedure and vacuum bell therapy. Patients and caregivers may not retain everything from one consultation, even after thorough counseling, and later LLM use may reinforce or clarify what the clinician discussed. After treatment, LLMs may also support standardized FAQs, discharge education, and follow-up guidance. These uses require responses that are accurate and complete as well as readable, understandable, actionable, and suited to patients and caregivers.

This study has several limitations. First, it included 30 standardized pediatric PE FAQs and only Simplified Chinese responses, so the results may not generalize to other questions, conditions, languages, cultures, or healthcare systems. Second, the single-turn protocol, character limit, and disabled web browsing, retrieval augmentation, and citation output improved comparability but did not represent personalized, multi-turn use. Third, we collected one response per question-model pair at a single time point. LLM outputs may vary even with identical prompts under similar conditions, so we could not assess within-model variability or establish whether model performance was stable and reproducible. Our findings are limited to the responses collected, and some differences between models may reflect this variability. Whether these differences would persist with repeated prompting or after model updates remains uncertain. Fourth, we used a study-specific adaptation of the C-PEMAT-P. The fixed 14-item form was prespecified and applied consistently across all models, but it has not been independently validated and is not directly equivalent to the original 24-item C-PEMAT-P. The three-item actionability component and the inclusion of many informational questions also limit the interpretation of between-model differences in actionability. Finally, anonymization and randomization reduced bias, but expert ratings remained partly subjective, and distinctive writing styles may have allowed reviewers to recognize some models. The panel included surgeons but not nurses, patients, or caregivers, whose priorities may differ. The effects of LLM-generated information on comprehension, trust, clinical decisions, and health behavior also remain uncertain. Future studies should examine more topics and languages, collect repeated outputs and real-world conversations, include multidisciplinary reviewers, and directly evaluate patients and caregivers.

## Conclusion

Leading LLMs generally generated accurate, complete, and relevant Simplified Chinese responses to common questions about pediatric PE, but no single model performed best across clinical content, readability, understandability, actionability, and overall quality. Claude achieved the highest accuracy, completeness, and GQS scores and ranked highest in GQS across all clinical domains, whereas DeepSeek showed the highest relevance. Kimi obtained the highest total C-PEMAT-P score, although *post hoc* testing distinguished it only from ChatGPT. Qwen showed the highest actionability but produced the least readable text and had the lowest understandability and overall quality. These findings indicate that factual accuracy alone is insufficient to characterize the quality of LLM-generated patient education materials and that multidimensional evaluation is necessary. Importantly, this study assessed LLM-generated materials using expert ratings and text-based evaluation tools rather than direct testing with pediatric patients or caregivers. Therefore, higher readability or understandability scores should not be interpreted as evidence of actual comprehension, trust, improved decision-making, or changes in health behavior. Future studies should directly evaluate patient and caregiver comprehension and other educational outcomes, including in repeated and multi-turn interactions and across broader clinical and linguistic settings.

## Data Availability

The original contributions presented in the study are included in the article/Supplementary material, further inquiries can be directed to the corresponding authors.

## Footnotes

1
https://github.com/corpustalk/AlphaReadabilityChinese2.0

2
http://www.chinesereadability.net/CRIE/?LANG=CHT#

## References

[1] Janssen N Coorens NA Franssen A Daemen JHT Michels IL Hulsewé KWE . Pectus excavatum and carinatum: a narrative review of epidemiology, etiopathogenesis, clinical features, and classification. J Thorac Dis. (2024) 16:1687–701. doi: 10.21037/jtd-23-957, 38505013 PMC10944748

[2] Scalise PN Demehri FR. The management of pectus excavatum in pediatric patients: a narrative review. Transl Pediatr. (2023) 12:208–20. doi: 10.21037/tp-22-361, 36891368 PMC9986778

[3] Notrica DM McMahon LE Jaroszewski DE. Pectus disorders: Excavatum, Carinatum and Arcuatum. Adv Pediatr Infect Dis. (2024) 71:181–94. doi: 10.1016/j.yapd.2024.05.001, 38944483

[4] Dunning J Burdett C Child A Davies C Eastwood D Goodacre T . The pectus care guidelines: best practice consensus guidelines from the joint specialist societies SCTS/MF/CWIG/BOA/BAPS for the treatment of patients with pectus abnormalities. Eur J Cardiothorac Surg. (2024) 66:ezae166. doi: 10.1093/ejcts/ezae166, 38964837

[5] Huo B Boyle A Marfo N Tangamornsuksan W Steen JP McKechnie T . Large language models for Chatbot health advice studies: a systematic review. JAMA Netw Open. (2025) 8:e2457879. doi: 10.1001/jamanetworkopen.2024.57879, 39903463 PMC11795331

[6] Tan J Wang L Wang G Yang Y Jia F Chi X . Safety and user perception of general-purpose large language models in pediatric healthcare: evaluations of ChatGPT by doctors and parents. Digit Health. (2026) 12:20552076261427505. doi: 10.1177/20552076261427505, 41836613 PMC12979900

[7] Sirzai EY Sezen CB Isik GO Yuksel M. Evaluating the accuracy and patient perception of AI-generated answers on pectus surgery. J Cardiothorac Surg. (2025) 20:437. doi: 10.1186/s13019-025-03669-3, 41291895 PMC12645766

[8] Oke D Illeez OG Giray E Ciftci B. Performance of ChatGPT-4o, Gemini 2.0 pro, and DeepSeek-V3 in patient-facing information on chest wall deformities: a comparative evaluation of accuracy, reliability, and reproducibility. Diagnostics. (2026) 16:589. doi: 10.3390/diagnostics16040589, 41750737 PMC12939082

[9] Zou X Lin Y Jin H Cai S Xu X Yin W . Screening for pectus excavatum among primary students and establishment of a pectus excavatum screening program in Dongguan, China. J Thorac Dis. (2015) 7:868–74. doi: 10.3978/j.issn.2072-1439.2015.04.48 PMC4454853 26101642

[10] Janssen N Daemen JHT van Polen EJ Coorens NA Jansen YJL Franssen A . Pectus excavatum: consensus and controversies in clinical practice. Ann Thorac Surg. (2023) 116:191–9. doi: 10.1016/j.athoracsur.2023.02.059 36997016

[11] Qiao G Mo X Zeng Q. National consensus on surgery for pectus excavatum in China. Chin J Pediatr Surg. (2020) 41:7–12. doi: 10.3760/cma.j.issn.0253-3006.2020.01.002

[12] Tam TYC Sivarajkumar S Kapoor S Stolyar AV Polanska K McCarthy KR . A framework for human evaluation of large language models in healthcare derived from literature review. NPJ Digit Med. (2024) 7:258. doi: 10.1038/s41746-024-01258-7, 39333376 PMC11437138

[13] Lei L Wei Y Liu K. AlphareadabilityChinese: a tool for the measurement of readability in Chinese texts and its applications. Foreign Lang Their Teach. (2024) 26:83–93. doi: 10.13458/j.cnki.flatt.004997

[14] Sung YT Chang TH Lin WC Hsieh KS Chang KE. CRIE: an automated analyzer for Chinese texts. Behav Res Methods. (2016) 48:1238–51. doi: 10.3758/s13428-015-0649-1, 26424442

[15] Shan Y Ji M Dong Z Xing Z Wang D Cao X. The Chinese version of the patient education materials assessment tool for printable materials: translation, adaptation, and validation study. J Med Internet Res. (2023) 25:e39808. doi: 10.2196/39808, 37200085 PMC10236277

[16] Bernard A Langille M Hughes S Rose C Leddin D Veldhuyzen van Zanten S. A systematic review of patient inflammatory bowel disease information resources on the world wide web. Am J Gastroenterol. (2007) 102:2070–7. doi: 10.1111/j.1572-0241.2007.01325.x, 17511753

[17] Zhang Y Wang L Zhang W Lan W. Decoupled quality and readability in skin cancer education from large language models. Front Public Health. (2026) 14:1777577. doi: 10.3389/fpubh.2026.1777577, 41799487 PMC12962940

[18] Zhong XF Liu R. Benchmarking the readability, quality, and educational suitability of large language models in communicating pertussis. Front Public Health. (2026) 14:1799204. doi: 10.3389/fpubh.2026.1799204, 42200119 PMC13199246

[19] Keçeci T Karagöz B. Can large language models follow guidelines? A comparative study of ChatGPT-4o and DeepSeek AI in clavicle fracture management based on AAOS recommendations. BMC Med Inform Decis Mak. (2025) 25:350. doi: 10.1186/s12911-025-03202-5, 41023674 PMC12482356

[20] Karagoz B Bayrak HC Keçeci T. Comparison of ChatGPT-5 and DeepSeek V3 for artificial intelligence-assisted patient education in foot and ankle disorders. J Am Podiatr Med Assoc. (2026) 116:116. doi: 10.3390/japma116030033, 42200996

[21] Liu R Chen Y Zhao W Cai Y. Performance evaluation of five major large language models in tuberculosis Q&a systems: a multidimensional assessment of readability, quality, and reliability. Digit Health. (2026) 12:20552076261467813. doi: 10.1177/20552076261467813, 42436893 PMC13354909

