# Supplementary material for: Comprehensive evaluation of large language models for pediatric pectus excavatum education
Source: Front Public Health. 2026 Oct 30;14:1990753. doi: 10.3389/fpubh.2026.1990753 (PMC13759771; doi:10.3389/fpubh.2026.1990753)
Supplement: Supplementary file 1 [file Table_1.docx]

Prompt in English:

Please answer the following question about pediatric pectus excavatum in Simplified Chinese, in a manner appropriate for affected children and their parents. Use clear, accessible, accurate, and objective language, and avoid unnecessary technical terminology. If technical terms are necessary, briefly explain them. Answer the question directly, and keep the response within 300 Chinese characters. Do not display references, citation numbers, DOIs, PMIDs, or web links, so as to maintain the natural style of a patient consultation. Do not use internet searches, web browsing, or any external tools. Question: [Insert question here]

Prompt in mandarin (Simplified Chinese):

请以面向患儿及其家长的方式，用简体中文回答以下关于儿童漏斗胸的问题。请使用清晰、通俗、准确且客观的语言，避免使用不必要的专业术语；如需使用专业术语，请进行简要解释。请直接回答问题，回答不得超过300个中文字符。请勿显示参考文献、引文编号、DOI、PMID或网页链接，以保持自然的患者咨询对话形式。不得使用联网搜索、网页浏览或任何外部工具。 问题：［在此插入问题］
