# Supplementary material for: Comprehensive evaluation of large language models for pediatric pectus excavatum education
Source: Front Public Health. 2026 Oct 30;14:1990753. doi: 10.3389/fpubh.2026.1990753 (PMC13759771; doi:10.3389/fpubh.2026.1990753)
Supplement: Supplementary file 2 [file Table_2.docx]

**Accuracy, completeness and relevance**

**Supplementary Table S1.** Overall evaluation of accuracy, completeness and relevance scores across the five LLMs

| **Metric** | **ChatGPT** | **Claude** | **DeepSeek** | **Kimi** | **Qwen** | **Kendall’s W (95% CI)** | **χ²** | **Adjusted P** |
| --- | --- | --- | --- | --- | --- | --- | --- | --- |
| Accuracy | 5.16 ± 0.34 | 5.39 ± 0.28 | 5.21 ± 0.25 | 5.08 ± 0.35 | 5.14 ± 0.32 | 0.12 (0.06–0.26) | 14.14 | **0.010*** |
| Completeness | 4.96 ± 0.34 | 5.22 ± 0.22 | 5.01 ± 0.33 | 5.12 ± 0.30 | 5.13 ± 0.40 | 0.12 (0.06–0.28) | 14.75 | **0.010*** |
| Relevance | 5.29 ± 0.27 | 5.35 ± 0.22 | 5.42 ± 0.27 | 5.27 ± 0.26 | 4.94 ± 0.37 | 0.33 (0.22–0.50) | 40.02 | **<0.001*** |

**Supplementary Table S2.** Pairwise comparisons of accuracy, completeness and relevance among the five LLMs

| **Metric** | **Comparison** | **Mean difference (95% CI)** | **Non-zero differences** | **Wilcoxon W** | **r_rb (95% CI)** | **Adjusted P** |
| --- | --- | --- | --- | --- | --- | --- |
| Accuracy | ChatGPT-Claude | -0.23 (-0.36, -0.11) | 24 | 48.00 | -0.68 (-0.93, -0.33) | **0.019*** |
|  | ChatGPT-DeepSeek | -0.05 (-0.18, 0.09) | 27 | 151.50 | -0.20 (-0.61, 0.22) | 1.000 |
|  | ChatGPT-Kimi | 0.07 (-0.08, 0.23) | 24 | 126.50 | 0.16 (-0.30, 0.58) | 1.000 |
|  | ChatGPT-Qwen | 0.01 (-0.15, 0.16) | 23 | 123.50 | 0.11 (-0.36, 0.56) | 1.000 |
|  | Claude-DeepSeek | 0.18 (0.07, 0.29) | 24 | 47.50 | 0.68 (0.33, 0.92) | **0.019*** |
|  | Claude-Kimi | 0.31 (0.19, 0.43) | 21 | 6.00 | 0.95 (0.81, 1.00) | **＜0.001*** |
|  | Claude-Qwen | 0.24 (0.09, 0.40) | 30 | 101.50 | 0.56 (0.20, 0.83) | **0.038*** |
|  | DeepSeek-Kimi | 0.12 (0.00, 0.26) | 22 | 75.50 | 0.40 (-0.05, 0.78) | 0.577 |
|  | DeepSeek-Qwen | 0.06 (-0.07, 0.19) | 25 | 129.00 | 0.21 (-0.25, 0.62) | 1.000 |
|  | Kimi-Qwen | -0.06 (-0.21, 0.09) | 29 | 174.50 | -0.20 (-0.61, 0.20) | 1.000 |
| Completeness | ChatGPT-Claude | -0.27 (-0.38, -0.14) | 28 | 54.00 | -0.73 (-0.97, -0.41) | **0.002^*^** |
|  | ChatGPT-DeepSeek | -0.06 (-0.20, 0.09) | 23 | 117.00 | -0.15 (-0.61, 0.32) | 1.000 |
|  | ChatGPT-Kimi | -0.16 (-0.32, 0.01) | 23 | 77.50 | -0.44 (-0.84, 0.01) | 0.465 |
|  | ChatGPT-Qwen | -0.18 (-0.34, -0.02) | 23 | 76.00 | -0.45 (-0.79, 0.00) | 0.465 |
|  | Claude-DeepSeek | 0.21 (0.08, 0.34) | 29 | 95.50 | 0.56 (0.20, 0.84) | 0.060 |
|  | Claude-Kimi | 0.11 (0.01, 0.21) | 22 | 69.00 | 0.45 (0.01, 0.81) | 0.465 |
|  | Claude-Qwen | 0.09 (-0.05, 0.23) | 25 | 126.00 | 0.22 (-0.24, 0.62) | 1.000 |
|  | DeepSeek-Kimi | -0.11 (-0.25, 0.04) | 27 | 130.50 | -0.31 (-0.68, 0.11) | 0.809 |
|  | DeepSeek-Qwen | -0.12 (-0.31, 0.07) | 29 | 164.00 | -0.25 (-0.64, 0.17) | 1.000 |
|  | Kimi-Qwen | -0.02 (-0.18, 0.14) | 26 | 167.00 | -0.05 (-0.50, 0.38) | 1.000 |
| Relevance | ChatGPT-Claude | -0.06 (-0.17, 0.06) | 27 | 158.00 | -0.16 (-0.57, 0.27) | 0.914 |
|  | ChatGPT-DeepSeek | -0.12 (-0.22, -0.02) | 24 | 80.00 | -0.47 (-0.83, -0.04) | 0.219 |
|  | ChatGPT-Kimi | 0.02 (-0.08, 0.13) | 26 | 156.50 | 0.11 (-0.34, 0.53) | 0.914 |
|  | ChatGPT-Qwen | 0.36 (0.23, 0.49) | 28 | 26.50 | 0.87 (0.66, 0.98) | **＜0.001*** |
|  | Claude-DeepSeek | -0.07 (-0.17, 0.04) | 20 | 72.00 | -0.31 (-0.76, 0.18) | 0.660 |
|  | Claude-Kimi | 0.08 (-0.02, 0.17) | 22 | 80.00 | 0.37 (-0.09, 0.81) | 0.509 |
|  | Claude-Qwen | 0.41 (0.28, 0.54) | 29 | 24.00 | 0.89 (0.71, 0.99) | **＜0.001*** |
|  | DeepSeek-Kimi | 0.14 (0.05, 0.24) | 25 | 70.50 | 0.57 (0.18, 0.89) | 0.053 |
|  | DeepSeek-Qwen | 0.48 (0.36, 0.60) | 26 | 3.00 | 0.98 (0.92, 1.00) | **＜0.001*** |
|  | Kimi-Qwen | 0.33 (0.19, 0.48) | 29 | 52.00 | 0.76 (0.48, 0.95) | **0.001*** |

**Supplementary Table S3.** Evaluation of accuracy, completeness and relevance across domains in five LLMs

| **Metric** | **Domain** | **ChatGPT** | **Claude** | **DeepSeek** | **Kimi** | **Qwen** | **Kendall’s W (95% CI)** | **χ²** | **Adjusted P** |
| --- | --- | --- | --- | --- | --- | --- | --- | --- | --- |
| Accuracy | Basic knowledge | 5.22 ± 0.40 | 5.28 ± 0.20 | 4.97 ± 0.22 | 5.06 ± 0.25 | 5.19 ± 0.36 | 0.12 (0.04–0.67) | 2.83 | 1.000 |
|  | Symptoms and Diagnosis | 5.31 ± 0.13 | 5.44 ± 0.20 | 5.25 ± 0.31 | 5.11 ± 0.47 | 5.11 ± 0.38 | 0.12 (0.04–0.64) | 2.80 | 1.000 |
|  | Observation and Conservative treatment | 5.11 ± 0.14 | 5.56 ± 0.25 | 5.33 ± 0.15 | 5.22 ± 0.20 | 5.03 ± 0.32 | 0.47 (0.29–0.83) | 11.20 | 0.122 |
|  | Surgical treatment | 4.97 ± 0.48 | 5.17 ± 0.24 | 5.19 ± 0.16 | 5.06 ± 0.34 | 5.22 ± 0.33 | 0.13 (0.05–0.59) | 3.15 | 1.000 |
|  | Prognosis and Precautions | 5.17 ± 0.41 | 5.50 ± 0.35 | 5.28 ± 0.25 | 4.97 ± 0.46 | 5.17 ± 0.30 | 0.28 (0.19–0.71) | 6.73 | 0.604 |
| Completeness | Basic knowledge | 4.97 ± 0.41 | 5.28 ± 0.20 | 4.83 ± 0.30 | 5.08 ± 0.23 | 5.33 ± 0.41 | 0.23 (0.07–0.73) | 5.46 | 0.781 |
|  | Symptoms and Diagnosis | 5.03 ± 0.29 | 5.25 ± 0.09 | 4.94 ± 0.51 | 5.17 ± 0.15 | 5.17 ± 0.47 | 0.21 (0.11–0.69) | 5.15 | 0.781 |
|  | Observation and Conservative treatment | 5.08 ± 0.17 | 5.33 ± 0.15 | 5.17 ± 0.15 | 5.28 ± 0.20 | 4.89 ± 0.17 | 0.46 (0.27–0.89) | 11.05 | 0.130 |
|  | Surgical treatment | 4.67 ± 0.24 | 5.03 ± 0.19 | 4.92 ± 0.25 | 5.00 ± 0.49 | 5.00 ± 0.54 | 0.25 (0.21–0.68) | 6.05 | 0.781 |
|  | Prognosis and Precautions | 5.03 ± 0.46 | 5.22 ± 0.31 | 5.19 ± 0.25 | 5.06 ± 0.31 | 5.28 ± 0.25 | 0.14 (0.09–0.65) | 3.28 | 0.781 |
| Relevance | Basic knowledge | 5.19 ± 0.36 | 5.31 ± 0.19 | 5.36 ± 0.27 | 5.17 ± 0.24 | 4.64 ± 0.13 | 0.56 (0.49–0.85) | 13.47 | **0.037*** |
|  | Symptoms and Diagnosis | 5.42 ± 0.35 | 5.39 ± 0.09 | 5.39 ± 0.17 | 5.42 ± 0.20 | 5.08 ± 0.35 | 0.17 (0.07–0.70) | 4.04 | 0.401 |
|  | Observation and Conservative treatment | 5.33 ± 0.15 | 5.47 ± 0.22 | 5.64 ± 0.16 | 5.44 ± 0.20 | 4.97 ± 0.40 | 0.60 (0.40–0.91) | 14.41 | **0.030*** |
|  | Surgical treatment | 5.19 ± 0.27 | 5.22 ± 0.33 | 5.19 ± 0.32 | 5.14 ± 0.36 | 4.92 ± 0.40 | 0.29 (0.19–0.72) | 6.93 | 0.280 |
|  | Prognosis and Precautions | 5.33 ± 0.15 | 5.36 ± 0.19 | 5.50 ± 0.26 | 5.19 ± 0.13 | 5.08 ± 0.43 | 0.41 (0.31–0.87) | 9.89 | 0.127 |

**CRIE**

**Supplementary Table S4.** Overall evaluation of CRIE grade levels across the five LLMs

|  | **ChatGPT** | **Claude** | **DeepSeek** | **Kimi** | **Qwen** | **Kendall’s W (95% CI)** | **χ²** | **P** |
| --- | --- | --- | --- | --- | --- | --- | --- | --- |
| CRIE grade M（Q1, Q3） | 5.5 (3, 6) | 6 (4, 6) | 6 (4, 7) | 6 (5, 6) | 7 (7, 8) | 0.36 (0.25–0.53) | 43.14 | **<0.001^*^** |
| Grade 1–6, n (%) | 24 (80.0%) | 23 (76.7%) | 20 (66.7%) | 26 (86.7%) | 7 (23.3%) |  |  |  |
| Grade 7–9, n (%) | 5 (16.7%) | 7 (23.3%) | 9 (30.0%) | 4 (13.3%) | 23 (76.7%) |  |  |  |
| Grade 10–12, n (%) | 1 (3.3%) | 0 (0.0%) | 1 (3.3%) | 0 (0.0%) | 0 (0.0%) |  |  |  |

**Supplementary Table S5.** Pairwise comparisons of CRIE grade levels among the five LLMs

|  | **Comparison** | **Mean difference (95% CI)** | **Non-zero differences** | **Wilcoxon W** | **r_rb (95% CI)** | **Adjusted P** |
| --- | --- | --- | --- | --- | --- | --- |
| CRIE grade | ChatGPT-Claude | -0.40 (-0.83, 0.07) | 23 | 86.00 | -0.38 (-0.76, 0.08) | 0.576 |
|  | ChatGPT-DeepSeek | -0.73 (-1.23, -0.17) | 22 | 56.50 | -0.55 (-0.90, -0.13) | 0.121 |
|  | ChatGPT-Kimi | -0.43 (-0.93, 0.07) | 22 | 82.00 | -0.35 (-0.78, 0.12) | 0.576 |
|  | ChatGPT-Qwen | -1.93 (-2.50, -1.37) | 26 | 9.00 | -0.95 (-1.00, -0.83) | **<0.001*** |
|  | Claude-DeepSeek | -0.33 (-0.87, 0.23) | 23 | 108.00 | -0.22 (-0.68, 0.24) | 0.848 |
|  | Claude-Kimi | -0.03 (-0.47, 0.37) | 18 | 84.00 | -0.02 (-0.52, 0.50) | 0.981 |
|  | Claude-Qwen | -1.53 (-2.00, -1.10) | 24 | 5.00 | -0.97 (-1.00, -0.86) | **<0.001*** |
|  | DeepSeek-Kimi | 0.30 (-0.17, 0.77) | 22 | 94.50 | 0.25 (-0.22, 0.71) | 0.848 |
|  | DeepSeek-Qwen | -1.20 (-1.73, -0.70) | 21 | 15.00 | -0.87 (-1.00, -0.64) | **<0.001*** |
|  | Kimi-Qwen | -1.50 (-1.90, -1.10) | 27 | 8.00 | -0.96 (-1.00, -0.85) | **<0.001*** |

**Supplementary Table S6.** Evaluation of CRIE grade levels across domains in five LLMs

| **Domain** | **Outcomes** | **ChatGPT** | **Claude** | **DeepSeek** | **Kimi** | **Qwen** | **Kendall’s W (95% CI)** | **χ²** | **Adjusted P** |
| --- | --- | --- | --- | --- | --- | --- | --- | --- | --- |
| Basic knowledge | CRIE grade, M (Q1, Q3) | 4.5 (4, 5) | 5.5 (4.3, 6) | 6 (6, 6) | 5.5 (5, 6) | 6.5 (6, 7.8) | 0.65 (0.58–0.87) | 15.50 | **0.019^*^** |
|  | Grade 1–6, n (%) | 6 (100.0%) | 6 (100.0%) | 5 (83.3%) | 5 (83.3%) | 3 (50.0%) |  |  |  |
|  | Grade 7–9, n (%) | 0 (0.0%) | 0 (0.0%) | 1 (16.7%) | 1 (16.7%) | 3 (50.0%) |  |  |  |
|  | Grade 10–12, n (%) | 0 (0.0%) | 0 (0.0%) | 0 (0.0%) | 0 (0.0%) | 0 (0.0%) |  |  |  |
| Symptoms and diagnosis | CRIE grade, M (Q1, Q3) | 6.5 (6, 7.8) | 7 (6.3, 7.8) | 7.5 (5.5, 8.8) | 6.5 (5.3, 7.8) | 7.5 (7, 8) | 0.25 (0.17–0.72) | 5.92 | 0.205 |
|  | Grade 1–6, n (%) | 3 (50.0%) | 2 (33.3%) | 2 (33.3%) | 3 (50.0%) | 0 (0.0%) |  |  |  |
|  | Grade 7–9, n (%) | 2 (33.3%) | 4 (66.7%) | 3 (50.0%) | 3 (50.0%) | 6 (100.0%) |  |  |  |
|  | Grade 10–12, n (%) | 1 (16.7%) | 0 (0.0%) | 1 (16.7%) | 0 (0.0%) | 0 (0.0%) |  |  |  |
| Observation and Conservative treatment | CRIE grade, M (Q1, Q3) | 5 (3.3, 6) | 5.5 (4.3, 6) | 6.5 (4.5, 7) | 5.5 (5, 6) | 7 (6.3, 7) | 0.45 (0.32–0.82) | 10.83 | 0.086 |
|  | Grade 1–6, n (%) | 6 (100.0%) | 5 (83.3%) | 3 (50.0%) | 6 (100.0%) | 2 (33.3%) |  |  |  |
|  | Grade 7–9, n (%) | 0 (0.0%) | 1 (16.7%) | 3 (50.0%) | 0 (0.0%) | 4 (66.7%) |  |  |  |
|  | Grade 10–12, n (%) | 0 (0.0%) | 0 (0.0%) | 0 (0.0%) | 0 (0.0%) | 0 (0.0%) |  |  |  |
| Surgical treatment | CRIE grade, M (Q1, Q3) | 6 (3.8, 6.8) | 5.5 (3.5, 6.8) | 5.5 (4.3, 6) | 6 (6, 6) | 7 (6.3, 7.8) | 0.33 (0.25–0.76) | 7.88 | 0.192 |
|  | Grade 1–6, n (%) | 4 (66.7%) | 4 (66.7%) | 5 (83.3%) | 6 (100.0%) | 2 (33.3%) |  |  |  |
|  | Grade 7–9, n (%) | 2 (33.3%) | 2 (33.3%) | 1 (16.7%) | 0 (0.0%) | 4 (66.7%) |  |  |  |
|  | Grade 10–12, n (%) | 0 (0.0%) | 0 (0.0%) | 0 (0.0%) | 0 (0.0%) | 0 (0.0%) |  |  |  |
| Prognosis and precautions | CRIE grade, M (Q1, Q3) | 3.5 (3, 5.5) | 5 (4.3, 5.8) | 4.5 (4, 5.8) | 4.5 (4, 5.8) | 7 (7, 7) | 0.60 (0.53–0.85) | 14.46 | **0.024^*^** |
|  | Grade 1–6, n (%) | 5 (83.3%) | 6 (100.0%) | 5 (83.3%) | 6 (100.0%) | 0 (0.0%) |  |  |  |
|  | Grade 7–9, n (%) | 1 (16.7%) | 0 (0.0%) | 1 (16.7%) | 0 (0.0%) | 6 (100.0%) |  |  |  |
|  | Grade 10–12, n (%) | 0 (0.0%) | 0 (0.0%) | 0 (0.0%) | 0 (0.0%) | 0 (0.0%) |  |  |  |

**ARC**

**Supplementary Table S7.** Pairwise comparisons of ARC readability metrics among the five LLMs

| **ARC metric** | **Comparison** | **Median paired difference (95% CI)** | **Non-zero differences** | **Wilcoxon W** | **r_rb (95% CI / limits)** | **Adjusted P** |
| --- | --- | --- | --- | --- | --- | --- |
| Lexical richness | ChatGPT-Claude | -0.56 (-0.64, -0.48) | 30 | 0.00 | -1.00 (-1.00, -1.00) | **<0.001*** |
|  | ChatGPT-DeepSeek | -0.35 (-0.43, -0.28) | 30 | 1.00 | -1.00 (-1.00, -0.97) | **<0.001*** |
|  | ChatGPT-Kimi | -0.56 (-0.62, -0.49) | 30 | 0.00 | -1.00 (-1.00, -1.00) | **<0.001*** |
|  | ChatGPT-Qwen | -1.01 (-1.14, -0.77) | 30 | 0.00 | -1.00 (-1.00, -1.00) | **<0.001*** |
|  | Claude-DeepSeek | 0.20 (0.12, 0.29) | 30 | 24.00 | 0.90 (0.70, 1.00) | **<0.001*** |
|  | Claude-Kimi | 0.01 (-0.08, 0.07) | 30 | 227.00 | 0.02 (-0.38, 0.43) | 0.919 |
|  | Claude-Qwen | -0.44 (-0.51, -0.29) | 30 | 9.00 | -0.96 (-1.00, -0.86) | **<0.001*** |
|  | DeepSeek-Kimi | -0.20 (-0.31, -0.12) | 30 | 16.00 | -0.93 (-1.00, -0.79) | **<0.001*** |
|  | DeepSeek-Qwen | -0.68 (-0.77, -0.56) | 30 | 0.00 | -1.00 (-1.00, -1.00) | **<0.001*** |
|  | Kimi-Qwen | -0.45 (-0.54, -0.35) | 30 | 4.00 | -0.98 (-1.00, -0.92) | **<0.001*** |
| Syntactic richness | ChatGPT-Claude | -0.06 (-0.10, -0.03) | 30 | 87.00 | -0.63 (-0.88, -0.29) | **0.014*** |
|  | ChatGPT-DeepSeek | -0.05 (-0.10, -0.02) | 30 | 101.00 | -0.57 (-0.87, -0.20) | **0.027*** |
|  | ChatGPT-Kimi | -0.10 (-0.14, -0.06) | 30 | 11.00 | -0.95 (-1.00, -0.85) | **<0.001*** |
|  | ChatGPT-Qwen | -0.11 (-0.13, -0.05) | 30 | 30.00 | -0.87 (-0.99, -0.67) | **<0.001*** |
|  | Claude-DeepSeek | -0.01 (-0.02, 0.05) | 30 | 207.00 | 0.11 (-0.33, 0.50) | 0.612 |
|  | Claude-Kimi | -0.06 (-0.08, 0.00) | 30 | 92.00 | -0.60 (-0.86, -0.26) | **0.018*** |
|  | Claude-Qwen | -0.03 (-0.06, -0.02) | 30 | 125.00 | -0.46 (-0.82, -0.07) | 0.079 |
|  | DeepSeek-Kimi | -0.05 (-0.09, -0.02) | 30 | 81.00 | -0.65 (-0.89, -0.32) | **0.010*** |
|  | DeepSeek-Qwen | -0.04 (-0.07, -0.01) | 30 | 100.00 | -0.57 (-0.87, -0.20) | **0.027*** |
|  | Kimi-Qwen | 0.01 (-0.01, 0.04) | 30 | 176.00 | 0.24 (-0.17, 0.62) | 0.507 |
| Semantic accuracy, noun | ChatGPT-Claude | -0.67 (-1.11, -0.33) | 30 | 37.00 | -0.84 (-1.00, -0.60) | **<0.001*** |
|  | ChatGPT-DeepSeek | -0.14 (-0.35, 0.01) | 30 | 146.00 | -0.37 (-0.74, 0.03) | 0.153 |
|  | ChatGPT-Kimi | -1.16 (-1.28, -0.66) | 29 | 33.00 | -0.85 (-0.99, -0.60) | **<0.001*** |
|  | ChatGPT-Qwen | -0.82 (-1.00, -0.24) | 30 | 78.00 | -0.66 (-0.92, -0.32) | **0.007*** |
|  | Claude-DeepSeek | 0.48 (0.10, 0.81) | 30 | 80.00 | 0.66 (0.33, 0.90) | **0.007*** |
|  | Claude-Kimi | -0.27 (-0.58, -0.11) | 30 | 130.00 | -0.44 (-0.79, -0.06) | 0.104 |
|  | Claude-Qwen | 0.05 (-0.21, 0.38) | 30 | 211.00 | 0.09 (-0.33, 0.49) | 0.670 |
|  | DeepSeek-Kimi | -0.77 (-1.24, -0.27) | 30 | 36.00 | -0.85 (-1.00, -0.62) | **<0.001*** |
|  | DeepSeek-Qwen | -0.54 (-0.71, -0.21) | 30 | 80.00 | -0.66 (-0.91, -0.33) | **0.007*** |
|  | Kimi-Qwen | 0.34 (0.06, 0.50) | 30 | 100.00 | 0.57 (0.22, 0.84) | **0.022*** |
| Semantic accuracy, verb | ChatGPT-Claude | 0.03 (-0.85, 0.47) | 30 | 169.00 | -0.27 (-0.63, 0.14) | 1.000 |
|  | ChatGPT-DeepSeek | 0.35 (-0.09, 0.55) | 30 | 174.00 | 0.25 (-0.17, 0.65) | 1.000 |
|  | ChatGPT-Kimi | -0.20 (-0.79, 0.37) | 30 | 172.00 | -0.26 (-0.63, 0.14) | 1.000 |
|  | ChatGPT-Qwen | -0.11 (-0.52, 0.00) | 30 | 135.00 | -0.42 (-0.75, -0.04) | 0.359 |
|  | Claude-DeepSeek | 0.26 (0.01, 0.72) | 30 | 138.00 | 0.41 (0.02, 0.75) | 0.366 |
|  | Claude-Kimi | -0.01 (-0.29, 0.45) | 30 | 230.00 | 0.01 (-0.40, 0.42) | 1.000 |
|  | Claude-Qwen | -0.10 (-0.54, 0.18) | 30 | 196.00 | -0.16 (-0.56, 0.26) | 1.000 |
|  | DeepSeek-Kimi | -0.34 (-0.66, -0.04) | 30 | 126.00 | -0.46 (-0.79, -0.09) | 0.250 |
|  | DeepSeek-Qwen | -0.42 (-0.62, -0.16) | 30 | 88.00 | -0.62 (-0.88, -0.28) | 0.022* |
|  | Kimi-Qwen | -0.09 (-0.30, 0.44) | 30 | 221.00 | -0.05 (-0.45, 0.38) | 1.000 |
| Semantic accuracy, noun–verb | ChatGPT-Claude | -0.43 (-0.90, 0.07) | 30 | 112.00 | -0.52 (-0.80, -0.15) | 0.060 |
|  | ChatGPT-DeepSeek | 0.10 (-0.11, 0.41) | 30 | 211.00 | 0.09 (-0.32, 0.51) | 1.000 |
|  | ChatGPT-Kimi | -0.58 (-0.96, -0.32) | 30 | 67.00 | -0.71 (-0.94, -0.42) | **0.002*** |
|  | ChatGPT-Qwen | -0.56 (-0.81, -0.11) | 30 | 100.00 | -0.57 (-0.87, -0.21) | **0.032*** |
|  | Claude-DeepSeek | 0.37 (0.22, 0.70) | 30 | 58.00 | 0.75 (0.45, 0.97) | **0.001*** |
|  | Claude-Kimi | -0.19 (-0.47, 0.10) | 30 | 156.00 | -0.33 (-0.69, 0.08) | 0.476 |
|  | Claude-Qwen | -0.15 (-0.46, 0.30) | 30 | 206.00 | -0.11 (-0.52, 0.29) | 1.000 |
|  | DeepSeek-Kimi | -0.73 (-0.91, -0.39) | 30 | 31.00 | -0.87 (-1.00, -0.66) | **<0.001*** |
|  | DeepSeek-Qwen | -0.49 (-0.81, -0.31) | 30 | 51.00 | -0.78 (-0.96, -0.52) | **<0.001*** |
|  | Kimi-Qwen | 0.07 (-0.09, 0.41) | 30 | 171.00 | 0.26 (-0.15, 0.63) | 0.639 |
| Semantic accuracy, content words | ChatGPT-Claude | -0.46 (-0.77, -0.02) | 30 | 102.00 | -0.56 (-0.83, -0.21) | **0.035*** |
|  | ChatGPT-DeepSeek | -0.06 (-0.22, 0.37) | 30 | 213.00 | 0.08 (-0.34, 0.48) | 1.000 |
|  | ChatGPT-Kimi | -0.32 (-0.70, -0.14) | 30 | 85.00 | -0.63 (-0.90, -0.31) | **0.012*** |
|  | ChatGPT-Qwen | -0.31 (-0.63, -0.07) | 30 | 101.00 | -0.57 (-0.86, -0.21) | **0.035*** |
|  | Claude-DeepSeek | 0.43 (0.25, 0.69) | 30 | 64.00 | 0.72 (0.42, 0.95) | **0.002*** |
|  | Claude-Kimi | -0.07 (-0.35, 0.22) | 30 | 202.00 | -0.13 (-0.53, 0.28) | 1.000 |
|  | Claude-Qwen | 0.01 (-0.08, 0.28) | 30 | 210.00 | 0.10 (-0.32, 0.49) | 1.000 |
|  | DeepSeek-Kimi | -0.59 (-0.73, -0.22) | 30 | 43.00 | -0.82 (-0.97, -0.57) | **<0.001*** |
|  | DeepSeek-Qwen | -0.30 (-0.53, -0.20) | 30 | 64.00 | -0.72 (-0.95, -0.42) | **0.002*** |
|  | Kimi-Qwen | 0.06 (-0.15, 0.39) | 30 | 187.00 | 0.20 (-0.22, 0.57) | 1.000 |
| Semantic richness, noun | ChatGPT-Claude | 0.02 (0.01, 0.05) | 30 | 119.00 | 0.49 (0.11, 0.80) | 0.111 |
|  | ChatGPT-DeepSeek | 0.01 (-0.02, 0.03) | 30 | 190.00 | 0.18 (-0.24, 0.57) | 1.000 |
|  | ChatGPT-Kimi | 0.04 (0.02, 0.05) | 30 | 90.00 | 0.61 (0.23, 0.95) | **0.023*** |
|  | ChatGPT-Qwen | 0.01 (-0.01, 0.02) | 30 | 196.00 | 0.16 (-0.26, 0.55) | 1.000 |
|  | Claude-DeepSeek | -0.02 (-0.04, 0.00) | 30 | 127.00 | -0.45 (-0.77, -0.07) | 0.147 |
|  | Claude-Kimi | 0.00 (-0.01, 0.02) | 30 | 223.00 | 0.04 (-0.36, 0.45) | 1.000 |
|  | Claude-Qwen | -0.02 (-0.03, 0.00) | 30 | 99.00 | -0.57 (-0.84, -0.23) | **0.035*** |
|  | DeepSeek-Kimi | 0.02 (0.01, 0.03) | 30 | 95.00 | 0.59 (0.24, 0.88) | **0.030*** |
|  | DeepSeek-Qwen | -0.02 (-0.03, 0.04) | 30 | 220.00 | 0.05 (-0.37, 0.45) | 1.000 |
|  | Kimi-Qwen | -0.02 (-0.03, -0.01) | 30 | 87.00 | -0.63 (-0.90, -0.29) | **0.020*** |
| Semantic clarity, noun | ChatGPT-Claude | 0.00 (0.00, 0.00) | 30 | 178.00 | 0.23 (-0.17, 0.62) | 1.000 |
|  | ChatGPT-DeepSeek | 0.00 (0.00, 0.00) | 30 | 219.00 | -0.06 (-0.45, 0.35) | 1.000 |
|  | ChatGPT-Kimi | 0.00 (0.00, 0.00) | 30 | 225.00 | 0.03 (-0.38, 0.44) | 1.000 |
|  | ChatGPT-Qwen | 0.00 (0.00, 0.00) | 30 | 154.00 | 0.34 (-0.06, 0.68) | 1.000 |
|  | Claude-DeepSeek | 0.00 (0.00, 0.00) | 30 | 165.00 | -0.29 (-0.66, 0.11) | 1.000 |
|  | Claude-Kimi | 0.00 (0.00, 0.00) | 30 | 180.00 | -0.23 (-0.61, 0.18) | 1.000 |
|  | Claude-Qwen | 0.00 (0.00, 0.00) | 30 | 218.00 | 0.06 (-0.35, 0.46) | 1.000 |
|  | DeepSeek-Kimi | 0.00 (0.00, 0.00) | 30 | 201.00 | 0.14 (-0.27, 0.53) | 1.000 |
|  | DeepSeek-Qwen | 0.00 (0.00, 0.00) | 30 | 162.00 | 0.30 (-0.10, 0.66) | 1.000 |
|  | Kimi-Qwen | 0.00 (0.00, 0.00) | 30 | 183.00 | 0.21 (-0.21, 0.59) | 1.000 |
| Semantic noise, noun | ChatGPT-Claude | -2.55 (-4.80, -1.26) | 30 | 93.00 | -0.60 (-0.90, -0.24) | **0.029*** |
|  | ChatGPT-DeepSeek | -1.56 (-3.78, -0.31) | 29 | 110.00 | -0.49 (-0.83, -0.11) | 0.152 |
|  | ChatGPT-Kimi | -1.99 (-6.57, -0.06) | 29 | 124.00 | -0.43 (-0.77, -0.04) | 0.259 |
|  | ChatGPT-Qwen | -4.80 (-6.20, -2.90) | 30 | 59.00 | -0.75 (-0.96, -0.45) | **0.002*** |
|  | Claude-DeepSeek | 1.19 (-1.66, 3.29) | 30 | 189.00 | 0.19 (-0.23, 0.57) | 1.000 |
|  | Claude-Kimi | 0.17 (-2.10, 3.01) | 30 | 232.00 | 0.00 (-0.40, 0.42) | 1.000 |
|  | Claude-Qwen | -1.54 (-5.14, 0.98) | 30 | 159.00 | -0.32 (-0.68, 0.09) | 0.674 |
|  | DeepSeek-Kimi | -2.51 (-3.23, 1.82) | 30 | 199.00 | -0.14 (-0.55, 0.27) | 1.000 |
|  | DeepSeek-Qwen | -3.01 (-3.94, -1.00) | 30 | 123.00 | -0.47 (-0.82, -0.09) | 0.164 |
|  | Kimi-Qwen | -1.52 (-5.42, 0.21) | 30 | 163.00 | -0.30 (-0.68, 0.11) | 0.674 |
| Coherence | ChatGPT-Claude | 0.03 (0.01, 0.04) | 30 | 100.00 | 0.57 (0.21, 0.87) | **0.032*** |
|  | ChatGPT-DeepSeek | 0.02 (-0.02, 0.05) | 30 | 178.00 | 0.23 (-0.17, 0.62) | 0.542 |
|  | ChatGPT-Kimi | 0.02 (0.00, 0.03) | 30 | 152.00 | 0.35 (-0.06, 0.72) | 0.384 |
|  | ChatGPT-Qwen | 0.05 (0.04, 0.07) | 30 | 45.00 | 0.81 (0.56, 0.97) | **<0.001*** |
|  | Claude-DeepSeek | -0.02 (-0.03, 0.01) | 30 | 151.00 | -0.35 (-0.69, 0.07) | 0.384 |
|  | Claude-Kimi | -0.01 (-0.03, 0.00) | 30 | 127.00 | -0.45 (-0.78, -0.07) | 0.147 |
|  | Claude-Qwen | 0.03 (0.01, 0.04) | 30 | 79.00 | 0.66 (0.32, 0.94) | **0.007*** |
|  | DeepSeek-Kimi | 0.00 (-0.02, 0.01) | 30 | 216.00 | -0.07 (-0.48, 0.34) | 0.746 |
|  | DeepSeek-Qwen | 0.03 (0.01, 0.06) | 30 | 55.00 | 0.76 (0.50, 0.95) | **<0.001*** |
|  | Kimi-Qwen | 0.04 (0.03, 0.06) | 30 | 43.00 | 0.82 (0.57, 0.97) | **<0.001*** |

**C-PEMAT-P and GQS**

**Supplementary Table S8.** Pairwise comparisons of C-PEMAT-P and GQS scores among the five LLMs

|  | **Comparison** | **Mean difference (95% CI)** | **Non-zero differences** | **Wilcoxon W** | **r_rb (95% CI / limits)** | **Adjusted P** |
| --- | --- | --- | --- | --- | --- | --- |
| **C-PEMAT-P** | ChatGPT-Claude | -0.50 (-0.87, -0.17) | 11 | 7.50 | -0.77 (-1.00, -0.28) | 0.144 |
|  | ChatGPT-DeepSeek | -0.47 (-0.77, -0.20) | 9 | 0.00 | -1.00 (-1.00, -1.00) | **0.035*** |
|  | ChatGPT-Kimi | -0.80 (-1.20, -0.43) | 12 | 0.00 | -1.00 (-1.00, -1.00) | **0.005*** |
|  | ChatGPT-Qwen | -0.67 (-1.17, -0.20) | 18 | 30.00 | -0.65 (-0.92, -0.21) | 0.079 |
|  | Claude-DeepSeek | 0.03 (-0.23, 0.33) | 7 | 13.00 | 0.07 (-0.80, 1.00) | 1.000 |
|  | Claude-Kimi | -0.30 (-0.57, -0.07) | 8 | 3.00 | -0.83 (-1.00, -0.33) | 0.281 |
|  | Claude-Qwen | -0.17 (-0.57, 0.20) | 15 | 48.00 | -0.20 (-0.69, 0.43) | 1.000 |
|  | DeepSeek-Kimi | -0.33 (-0.63, -0.07) | 10 | 8.00 | -0.71 (-1.00, -0.14) | 0.283 |
|  | DeepSeek-Qwen | -0.20 (-0.63, 0.20) | 16 | 51.00 | -0.25 (-0.71, 0.35) | 1.000 |
|  | Kimi-Qwen | 0.13 (-0.23, 0.43) | 15 | 43.00 | 0.28 (-0.28, 0.86) | 1.000 |
| **GQS** | ChatGPT-Claude | -0.57 (-0.73, -0.40) | 17 | 0.00 | -1.00 (-1.00, -1.00) | **＜0.001*** |
|  | ChatGPT-DeepSeek | 0.43 (0.23, 0.63) | 12 | 0.00 | 1.00 (1.00, 1.00) | **0.001*** |
|  | ChatGPT-Kimi | 0.23 (-0.03, 0.53) | 11 | 16.50 | 0.50 (-0.09, 1.00) | 0.292 |
|  | ChatGPT-Qwen | 1.13 (0.83, 1.43) | 27 | 10.50 | 0.94 (0.81, 1.00) | **＜0.001*** |
|  | Claude-DeepSeek | 1.00 (0.80, 1.20) | 25 | 0.00 | 1.00 (1.00, 1.00) | **＜0.001*** |
|  | Claude-Kimi | 0.80 (0.57, 1.07) | 20 | 0.00 | 1.00 (1.00, 1.00) | **＜0.001*** |
|  | Claude-Qwen | 1.70 (1.43, 1.97) | 28 | 0.00 | 1.00 (1.00, 1.00) | **＜0.001*** |
|  | DeepSeek-Kimi | -0.20 (-0.40, 0.00) | 12 | 19.50 | -0.50 (-1.00, 0.00) | 0.292 |
|  | DeepSeek-Qwen | 0.70 (0.43, 1.00) | 18 | 7.50 | 0.91 (0.70, 1.00) | **＜0.001*** |
|  | Kimi-Qwen | 0.90 (0.67, 1.13) | 21 | 0.00 | 1.00 (1.00, 1.00) | **＜0.001*** |

**Supplementary Table S9.** Pairwise comparisons of understandability and actionability among the five LLMs

| **Metric** | **Comparison** | **Mean difference (95% CI)** | **Non-zero differences** | **Wilcoxon W** | **r_rb (95% CI / limits)** | **Adjusted P** |
| --- | --- | --- | --- | --- | --- | --- |
| Understandability | ChatGPT-Claude | -0.10 (-0.23, 0.00) | 3 | 0.00 | -1.00 (-1.00, -1.00) | 1.000 |
|  | ChatGPT-DeepSeek | -0.17 (-0.33, -0.03) | 4 | 0.00 | -1.00 (-1.00, -1.00) | 0.625 |
|  | ChatGPT-Kimi | -0.20 (-0.40, -0.07) | 5 | 0.00 | -1.00 (-1.00, -1.00) | 0.375 |
|  | ChatGPT-Qwen | 0.43 (0.23, 0.63) | 15 | 8.00 | 0.87 (0.56, 1.00) | **0.007*** |
|  | Claude-DeepSeek | -0.07 (-0.17, 0.00) | 2 | 0.00 | -1.00 (-1.00, -1.00) | 1.000 |
|  | Claude-Kimi | -0.10 (-0.23, 0.00) | 3 | 0.00 | -1.00 (-1.00, -1.00) | 1.000 |
|  | Claude-Qwen | 0.53 (0.33, 0.73) | 18 | 9.50 | 0.89 (0.62, 1.00) | **0.001*** |
|  | DeepSeek-Kimi | -0.03 (-0.20, 0.10) | 5 | 6.00 | -0.20 (-1.00, 1.00) | 1.000 |
|  | DeepSeek-Qwen | 0.60 (0.37, 0.83) | 18 | 8.50 | 0.90 (0.67, 1.00) | **0.001*** |
|  | Kimi-Qwen | 0.63 (0.40, 0.87) | 19 | 9.00 | 0.91 (0.68, 1.00) | **<0.001*** |
| Actionability | ChatGPT-Claude | -0.40 (-0.73, -0.07) | 9 | 6.00 | -0.73 (-1.00, -0.17) | 0.215 |
|  | ChatGPT-DeepSeek | -0.30 (-0.57, -0.07) | 5 | 0.00 | -1.00 (-1.00, -1.00) | 0.250 |
|  | ChatGPT-Kimi | -0.60 (-0.93, -0.30) | 10 | 0.00 | -1.00 (-1.00, -1.00) | **0.012*** |
|  | ChatGPT-Qwen | -1.10 (-1.53, -0.70) | 17 | 0.00 | -1.00 (-1.00, -1.00) | **<0.001*** |
|  | Claude-DeepSeek | 0.10 (-0.20, 0.40) | 6 | 8.00 | 0.24 (-0.67, 1.00) | 0.688 |
|  | Claude-Kimi | -0.20 (-0.43, 0.00) | 5 | 1.50 | -0.80 (-1.00, 0.00) | 0.375 |
|  | Claude-Qwen | -0.70 (-1.03, -0.40) | 16 | 6.00 | -0.91 (-1.00, -0.68) | **0.003*** |
|  | DeepSeek-Kimi | -0.30 (-0.57, -0.07) | 7 | 2.00 | -0.86 (-1.00, -0.33) | 0.250 |
|  | DeepSeek-Qwen | -0.80 (-1.23, -0.40) | 15 | 5.00 | -0.92 (-1.00, -0.68) | **0.004*** |
|  | Kimi-Qwen | -0.50 (-0.77, -0.27) | 12 | 0.00 | -1.00 (-1.00, -1.00) | **0.004*** |
